# Vitamin D Regulates Olfactory Function via Dual Transcriptional and mTOR‐Dependent Translational Control of Synaptic Proteins

**DOI:** 10.1002/advs.202507181

**Published:** 2025-12-16

**Authors:** Pengcheng Ren, Renhe Cao, Xiaoshan Ye, Wenbin Pang, Qingshang Bi, Meihui Huang, Qionglin Zhou, Dan Ye, Wei Xiang, Le Xiao

**Affiliations:** ^1^ Hainan Women and Children's Medical Center School of Pediatrics Hainan Medical University Hainan Academy of Medical Sciences Haikou 571199 China; ^2^ School of Basic Medicine and Life Science Hainan Medical University Hainan Academy of Medical Sciences Haikou 571199 China; ^3^ Present address: Sir Run Run Shaw Hospital School of Medicine Zhejiang University Hangzhou China; ^4^ Present address: Neonatology Department of Hainan General Hospital Hainan Academy of Medical Sciences Hainan Medical University Haikou China

**Keywords:** mTOR, olfactory bulb, olfactory function, synapse, translation, vitamin D, vitamin D receptor

## Abstract

Vitamin D (VitD) deficiency is associated with neurological dysfunction, but its cell‐type‐specific mechanisms remain poorly understood. Using mice with controlled VitD levels from weaning through adulthood, it is demonstrated that VitD regulates olfactory function through vitamin D receptors (VDR). Deficiency impairs odor discrimination, whereas supplementation enhances sensitivity—phenotypes recapitulated by olfactory‐specific VDR knockdown. Single‐nucleus RNA sequencing (snRNA‐seq) and spatial transcriptomics reveal enriched VDR expression selectively in olfactory bulb tufted cells, where VitD signaling mediates dendrodendritic synaptic remodeling via both transcriptional and translational mechanisms in a VDR‐dependent manner. Notably, VitD modulates synaptic protein expression partly through mechanistic target of rapamycin (mTOR) signaling, and rapamycin treatment restores translational homeostasis and olfactory function in VitD‐deficient mice. Chromatin immunoprecipitation sequencing (ChIP‐seq) confirms direct VDR binding to genes encoding synaptic proteins and translational machinery components, including mTOR pathway effectors. Together, these results identify a novel VDR‐mTOR‐translational regulatory axis that operates alongside classical transcriptional regulation, establishing VitD as a diet‐sensitive neuromodulator that links nutritional status to synaptic function and sensory processing.

## Graphical abstract

Vitamin D (VitD) modulates olfactory function by remodeling dendrodendritic synapses in tufted cells through vitamin D receptor‐dependent transcriptional and translational mechanisms. VitD regulates synaptic protein translation partially via mTOR signaling. Rapamycin rescues VitD deficiency‐induced olfactory deficits by restoring mTOR‐mediated translation, identifying VitD as a diet‐sensitive neuromodulator linking nutrition to synaptic function and sensory processing.

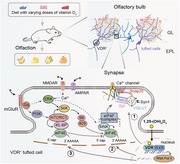

## 1 Introduction

Vitamin D (VitD) deficiency is a globally prevalent medical condition, affecting over 1 billion children and adults worldwide.^[^ ^1^ ^]^ While traditionally recognized for its function in regulating the bone metabolism, emerging research has elucidated broader physiological roles of VitD, particularly in the central nervous system.^[^ ^2^, ^3^ ^]^ VitD deficiency has been increasingly associated with a spectrum of brain dysfunctions, including sensory impairment,^[^ ^4^ ^]^ addiction,^[^ ^5^ ^]^ depression,^[^ ^6^, ^7^ ^]^ and neurodevelopmental disorders.^[^ ^8^, ^9^ ^]^ Consequently, VitD supplementation has been investigated as both a standalone or adjunctive therapeutic strategy for these neurological and psychiatric conditions, but the efficacy of such interventions remained inconclusive.^[^ ^10^, ^11^, ^12^, ^13^, ^14^ ^]^ Therefore, it is crucial to understand the mechanistic roles of VitD in the nervous system to fully unlock its pharmacological potential.

VitD, a fat‐soluble vitamin, exists in two primary forms: VitD_2_ (ergocalciferol) and VitD_3_ (cholecalciferol). Among these, VitD_3_ is physiologically more relevant due to its higher potency, longer half‐life, and greater abundance in animal‐based foods.^[^ ^2^ ^]^ Both VitD_2_ and D_3_ undergo hepatic hydroxylation in vivo to form 25‐hydroxyvitamin D_2_ [25(OH)D_2_] and 25‐hydroxyvitamin D_3_ [25(OH)D_3_], respectively. These 25‐hydroxylated metabolites represent the major circulating forms of VitD and serve as the standard biomarkers for assessing VitD status in clinical and research settings.^[^ ^15^ ^]^ Subsequent renal hydroxylation converts these metabolites into 1,25‐dihydroxyvitamin D_3_ [1,25(OH)_2_D_3_], the biologically active form characterized by a short half‐life and blood‐brain barrier permeability.^[^ ^16^ ^]^ The active metabolite 1,25(OH)_2_D_3_ mediates its effects primarily through binding to vitamin D receptors (VDRs).

VDRs are nuclear proteins that regulate gene expression by binding to specific DNA sequences.^[^ ^17^ ^]^ In the brain, VDRs are widely distributed across regions, including olfactory bulb (OB), cerebral cortex, hypothalamus, hippocampus and cerebellum.^[^ ^18^, ^19^ ^]^ This broad distribution highlights the potential significance of VitD in brain function but presents challenges in elucidating its region‐ and cell‐type‐specific effects. Furthermore, VitD has been implicated in the regulation of synaptic proteins, influencing key components of synaptic function such as presynaptic release machinery, neurotransmitter transporters, and receptors.^[^ ^20^, ^21^, ^22^, ^23^ ^]^ The expression and function of these synaptic proteins are highly context‐dependent, shaped by the specific neural circuits in which they are embedded and the synaptic roles they mediate.^[^ ^24^ ^]^

Although the synaptic regulation of VitD, inherently linked to VDR's function as a transcription factor, have been extensively studied,^[^ ^25^ ^]^ recent attention has turned to its roles in translational control with the nervous system. For instance, a study analyzing the SFARI Gene database revealed interconnections between VitD‐sensitive genes and the mechanistic target of rapamycin (mTOR) signaling pathway, an important controller of protein synthesis.^[^ ^26^, ^27^ ^]^ Additionally, phosphatase and tensin homolog (PTEN), a key regulator of mTOR signaling, has been implicated in VitD's effects; VitD supplementation reduces social impairments in *PTEN* knockout mice,^[^ ^28^ ^]^ suggesting a link between VitD and mTOR‐dependent protein synthesis. However, whether VitD regulates synaptic protein synthesis through mTOR‐mediated translational control, and how it is executed in a cell‐type‐specific manners to impact synapses and behaviors, remains unexplored.

We investigated VitD‐mediated regulation of olfactory function using a murine model with strictly controlled VitD status from weaning through early adulthood. Our findings reveal that VitD status directly modulates olfactory sensitivity: dietary deprivation impaired odor discrimination, whereas supplementation enhanced sensory acuity. Single‐nucleus RNA sequencing (snRNA‐seq) and spatial transcriptomic analyses identified predominant VDR expression in a specific tufted cell population within the OB, where it drives cell‐type‐specific remodeling of dendrodendritic synapses. VitD signaling was found to regulate both synaptic and translational pathways, with VDR knockdown phenocopying the olfactory impairments and synaptic/translational dysregulation observed in VitD‐deficient mice. Mechanistically, we demonstrated that VitD modulates synaptic protein expression partly through mTOR signaling‐mediated translational regulation, and rapamycin treatment restores translational homeostasis and olfactory function in VitD‐deficient mice. Chromatin immunoprecipitation sequencing (ChIP‐seq) further demonstrated that VDR directly binds to and regulates genomic loci encoding synaptic proteins and translational machinery components, including mTOR signaling effectors, supporting a mechanism of coordinated transcriptional and translational regulation. Together, these results identify a novel VDR‐mTOR‐translational regulatory axis that operates alongside classical transcriptional regulation. Our findings not only expand the understanding of VitD's neuromodulatory functions beyond classical endocrine roles but also suggest potential therapeutic applications targeting nutrient‐sensitive pathways in neurological disorders.

## 2 Results

### 2.1 Dietary Vitamin D (VitD) Levels Modulate Olfactory Function in Mice—Deficiency Impairs Odor Discrimination, whereas Supplementation Enhances Sensitivity

Since VDRs are the primary mediator of VitD's downstream signaling pathways, we first assessed the VDR expression at both mRNA and protein levels in the OB, cortex, hippocampus, thalamus and cerebellum. We found that the OB exhibited the highest level of VDR expression among these brain regions (Figure S1A, Supporting Information, *P* < 0.001, one‐way ANOVA with Tukey's multiple comparisons post hoc test). Furthermore, VDR expression was low at embryonic day 16 (E16) but increased postnatally, reaching consistently high levels by postnatal day 14 (P14, one‐way ANOVA with Tukey's multiple comparisons post hoc test) and persisting into two‐month‐old mice (Figure S1B, Supporting Information). These findings suggest that VitD, acting through VDRs, may significantly influence olfaction from childhood through early adulthood.

**Figure 1:**
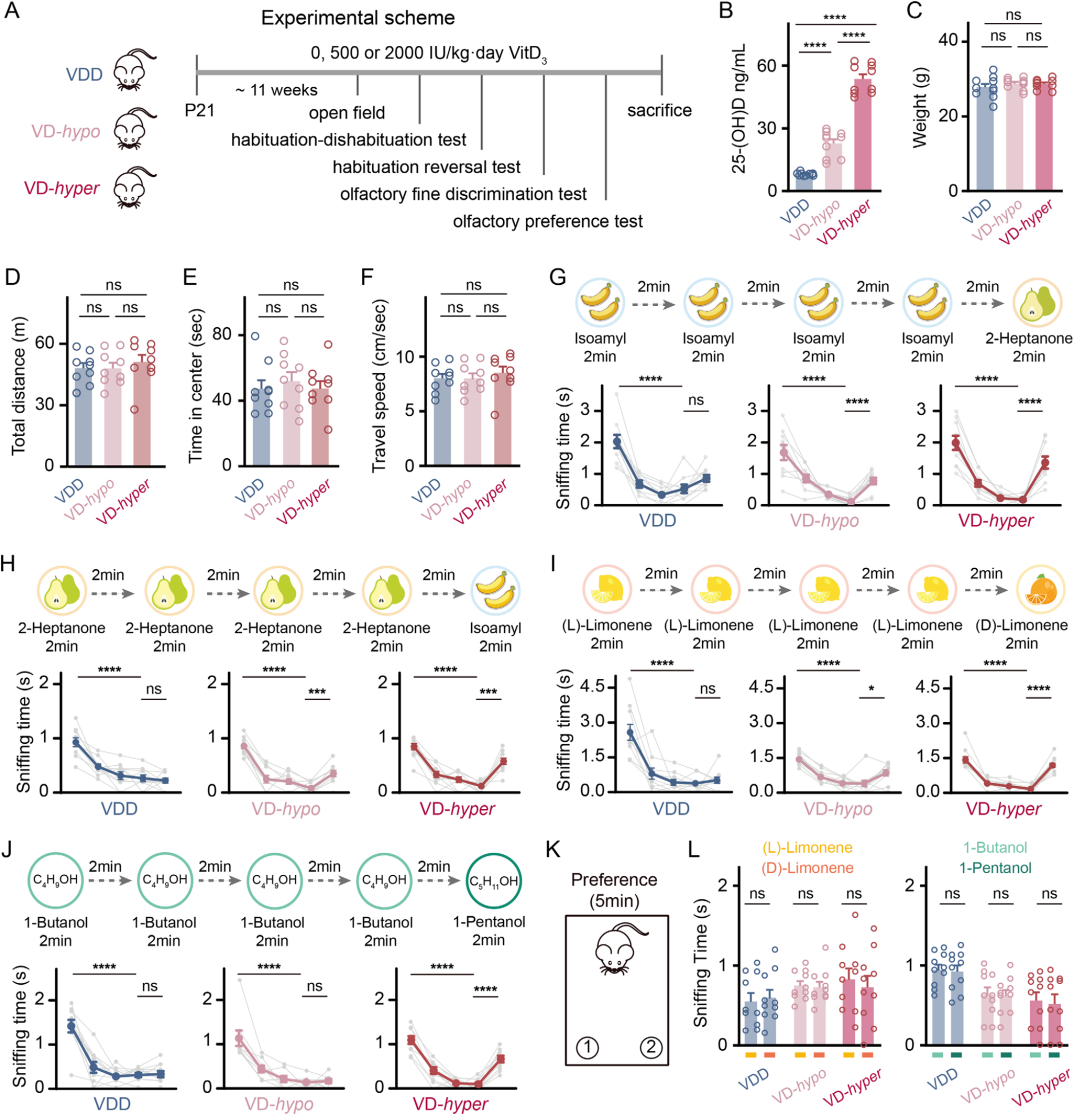
Dietary VitD levels modulate olfactory function in mice—deficiency impairs odor discrimination, whereas supplementation enhances sensitivity. A) Experimental design for behavioral tests conducted on mice fed with varying doses of VitD_3_ from weaning: 0 IU kg^−1^·day^−1^ (VDD group), 500 IU kg^−1^·day^−1^ (VD‐*hypo* group), and 2000 IU kg^−1^·day^−1^ (VD‐*hyper* group). B,C) Serum 25‐(OH)D levels (B) and body weights (C) of mice at 14 weeks of age (VDD, VD‐*hypo*, and VD‐*hyper* groups). D–F) Total distance traveled (D), time to enter the middle area (E), and average travel speed (F) of mice in the open field test. G–J) Sniffing time in the olfactory habituation‐dishabituation test (G, I, J) and olfactory habituation reversal test (H). Odor pairs were: isoamyl acetate and 2‐heptanone (G and H), (L)‐limonene and (D)‐limonene (I), and 1‐butanol and 1‐pentanol (J). K) Schematic diagram of the olfactory preference test. L) Sniffing time for (L)‐limonene versus (D)‐limonene (left panel) and 1‐butanol versus 1‐pentanol (right panel) in the olfactory preference test. Symbols = biological replicates; bars = mean ± SEM; *n* = 9–10 mice per group. One‐way repeated ANOVA followed by Tukey's multiple comparisons test was used for comparisons among trials of olfactory tests within one group, and also among three groups. The unpaired t‐test was used for the olfactory preference test. Significance levels: *P < 0.05, **P < 0.01, ***P < 0.001, ****P < 0.0001; ns: not significant.

Clinical observations have suggested that VitD deficiency is associated with the olfactory dysfunction, which can be alleviated by VitD supplementation in some patients.^[^ ^4^, ^29^, ^30^, ^31^, ^32^ ^]^ To investigate whether varying serum VitD levels affect olfaction in mice, we employed an animal model with a controlled gradient of dietary VitD intake from childhood to early adulthood, as previously described^[^ ^20^ ^]^ (**Figure** **1**A). In this model, mice were fed one of three diets based on AIN‐93G standard chow, each containing varying dosage of vitamin D_3_ (VitD_3_), starting at weaning (P21) and continuing for ≈11 weeks. The diets included: 0 IU kg^−1^·day^−1^ VitD_3_ (VitD deficiency, VDD), 500 IU kg^−1^·day^−1^ VitD_3_ (moderate VitD supplementation, VD‐*hypo*), and 2000 IU kg^−1^·day^−1^ VitD_3_ (high VitD supplementation, VD‐*hyper*). For accurate quantification, serum levels of 25‐(OH)D_2_ and 25‐(OH)D_3_ in these mice were measured using liquid chromatography‐mass spectrometry (LC‐MS) (Figure S2A,B, Supporting Information). And the average serum 25‐(OH)D levels, calculated as the sum of 25‐(OH)D_2_ and 25‐(OH)D_3_ levels, were significantly different between the groups (8.12 ± 0.24 ng mL^−1^ for VDD, 22.78 ± 1.99 ng mL^−1^ for VD‐*hypo* and 53.63 ± 2.22 ng mL^−1^ for VD‐*hyper*) (Figure 1B, *P* < 0.0001, one‐way ANOVA with Tukey's multiple comparisons post hoc test). In contrast, no significant differences were observed in the average body weight, body length, or brain weight across the groups (Figure 1C; Figure S2C,D, Supporting Information, one‐way ANOVA with Tukey's multiple comparisons post hoc test). Consistent with prior findings,^[^ ^20^ ^]^ 25‐(OH)D concentrations in the VD‐*hypo* group exhibited comparable but marginally elevated levels relative to control animals maintained on standard AIN‐93G diet under ad libitum feeding conditions (19.77 ± 1.01 ng mL^−1^ for AIN‐93G, *P* < 0.01, unpaired t‐test). Additionally, gross morphology of the OB, cerebral cortex, hippocampus and third ventricle revealed no significant changes associated with varying VitD intake (Figure S2E–N, Supporting Information, one‐way ANOVA with Tukey's multiple comparisons post hoc test).

Next, we conducted a series of behavioral experiments to assess basic motor function and olfactory function in the three groups of mice. In the open field test, all groups exhibited comparable performance in terms of total distance traveled, time spent in the center, and travel speed (Figure 1D–F, one‐way ANOVA with Tukey's multiple comparisons post hoc test). These findings suggest that the gradient of serum 25(OH)D levels, resulting from differential dietary VitD intake, did not affect the general motor functions in the mice.

We subsequently evaluated olfactory function using a habituation‐dishabituation paradigm. Mice were initially exposed to four trials of isoamyl acetate, which emits a banana‐like scent, followed by a switch to 2‐heptanone, which has a pear‐like scent, in the fifth trial. All groups demonstrated a significant decrease in sniffing time between the first and fourth presentations of isoamyl acetate (Figure 1G, *P *< 0.0001, one‐way repeated ANOVA with Tukey's multiple comparisons post hoc test). However, both the VD‐*hypo* and VD‐*hyper* groups showed a significant increase in sniffing time upon odor renewal in the fifth trial (Figure 1G, *P* < 0.0001, one‐way repeated ANOVA with Tukey's multiple comparisons post hoc test), whereas the VDD group did not (Figure 1G, one‐way repeated ANOVA with Tukey's multiple comparisons post hoc test). To control for potential non‐specific effects of the odors, we reversed the odor presentation sequence and repeated the test. Consistent with the initial results, the VDD group again showed no significant difference in sniffing time between the fifth trial and fourth trials, while both the VD‐*hypo* and VD‐*hyper* groups displayed significant increases (Figure 1H, *P* < 0.001, one‐way repeated ANOVA with Tukey's multiple comparisons post hoc test). These results indicate that VitD deficiency impairs olfactory function.

Next, we assessed the ability of the mice to discriminate between two similar odors in the habituation‐dishabituation test. Mice in both the VD‐*hypo* and VD‐*hyper* groups showed significantly increased sniffing time at the fifth trials when the odor changed from (L)‐limonene to (D)‐limonene (Figure 1I, *P* < 0.05 for VD‐*hypo*, *P* < 0.0001 for VD‐*hyper*, one‐way repeated ANOVA with Tukey's multiple comparisons post hoc test). In contrast, the sniffing time of VDD mice on the fifth trials was similar to the fourth trial (Figure 1I, one‐way repeated ANOVA with Tukey's multiple comparisons post hoc test). This suggests that VitD supplementation, regardless of dosage, enabled the mice to discriminate between the similar odors of (L)‐limonene to (D)‐limonene.

We then increased the difficulty of discrimination by presenting two highly similar odors, 1‐butanol and 1‐pentanol. While all three groups exhibited habituation during the first four trials with 1‐butanol (Figure 1J, *P* < 0.0001 for VDD, VD‐*hypo*, and VD‐*hyper*, one‐way repeated ANOVA with Tukey's multiple comparisons post hoc test), only the VD‐*hyper* group showed a significant increase in sniffing when presented with 1‐pentanol on the fifth trial (Figure 1J, *P* < 0.0001, one‐way repeated ANOVA with Tukey's multiple comparisons post hoc test). This indicates that high‐dose VitD intake improves the sensitivity in odor discrimination.

As a control, we assessed the mice's preference for the two pairs of odors ((L)‐ and (D)‐limonene; 1‐pentanol and 1‐butanol) and found no significant differences between the two odors within each pair (Figure 1K,L, unpaired t‐test). This confirms that the dishabituation observed on the fifth trial was due to the mice's detection of a new odor, rather than an intrinsic preference for that odor.

In a separate cohort, we tested the olfactory behaviors of mice fed a standard AIN‐93G diet ad libitum (Figure S3A, Supporting Information). The performance of the standard diet group (SD group) was similar to the VD‐*hypo* group. They were able to discriminate between isoamyl and 2‐heptanone, as well as (L)‐limonene and (D)‐limonene, but not between 1‐butanol and 1‐pentanol (Figure S3B–E, Supporting Information). Moreover, they showed similar preferences for the two pairs of odors ((L)‐limonene versus (D)‐limonene, and 1‐butanol versus 1‐pentanol) (Figure S3F,G, Supporting Information). Notably, serum 25(OH)D levels in the standard diet (SD) group at 18 weeks (24.47 ± 0.58 ng mL^−1^) were significantly higher than those previously observed at 15 weeks in our earlier study^[^ ^20^ ^]^ (19.77 ± 1.01 ng mL^−1^, *P* < 0.01, unpaired t‐test), but remained similar to levels in the VD‐*hypo* group at 15 weeks (22.78 ± 1.99 ng mL^−1^, *P* = 0.97, unpaired t‐test). These observations suggest that serum VitD levels increase with age during mouse adulthood. Notably, serum 25(OH)D concentrations and associated olfactory function in VD‐*hypo* mice remain physiologically comparable to those of mice maintained on a standard diet.

In summary, using a mouse model with gradient dietary VitD intake, we found that VitD deficiency or supplementation from childhood to early adulthood did not lead to morphological changes in the OB. However, it significantly impacted olfactory function. VitD‐deficient mice exhibited impairments in olfactory habituation‐dishabituation and fine odor discrimination. In contrast, mice receiving VitD supplementation showed enhanced abilities in fine odor discrimination, in a dose‐dependent manner.

### 2.2 Vitamin D Receptor (VDR) Knockdown in the Olfactory Bulb (OB) Impairs Olfactory Function in Mice

**Figure 2:**
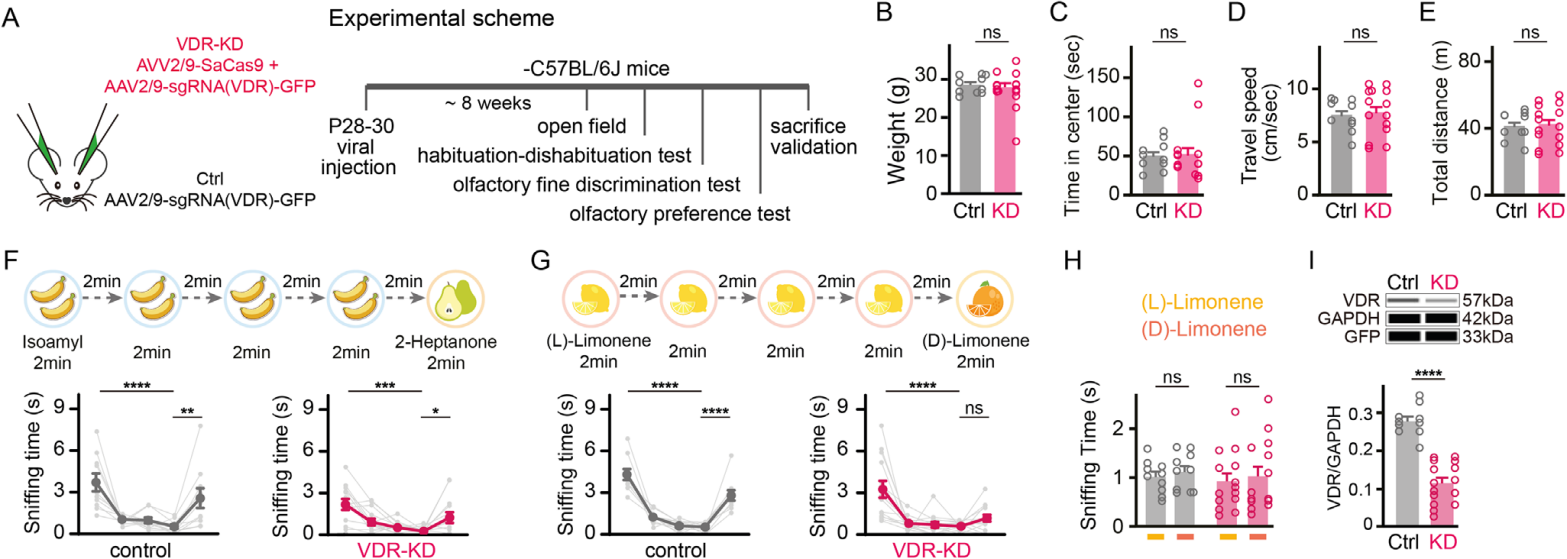
VDR knockdown in the OB impairs olfactory function in mice. A) Experimental schematic of SaCas9‐mediated VDR‐KD in the mouse OB and subsequent behavioral tests. B–E) Body weight (B) and measurements from the open field test, including time to enter the center area (C), average travel speed (D), and total distance traveled (E), in VDR‐KD and control groups (unpaired t‐test). F,G) Olfactory habituation‐dishabituation tests: sniffing time of VDR‐KD and control mice in response to odor pairs, including isoamyl acetate versus 2‐heptanone (F) and (L)‐limonene versus (D)‐limonene (G) (one‐way repeated ANOVA with Tukey's multiple comparisons). H) Sniffing time for (L)‐limonene and (D)‐limonene in the olfactory preference test for VDR‐KD and control groups (unpaired t‐test). I) The quantitative western blot (*wes*) image (upper panel) and quantification of VDR expression in VDR‐KD and control (Ctrl) groups (lower panel), measured after completion of behavioral tests (unpaired t‐test). Symbols = biological replicates; bars = mean ± SEM; *n* = 10 for control, *n* = 14 for VDR‐KD. Significance levels: *P < 0.05, **P < 0.01, ***P < 0.001, ****P < 0.0001; ns: not significant.

Given that VitD‐deficient mice exhibit impaired olfactory function and considering the high expression of the VDR in the OB, we next investigated whether OB‐expressing VDR mediated VitD‐dependent olfactory functions. To address this, we knocked down VDR expression in the OB of C57BL/6 mice using adeno‐associated virus (AAV) vectors encoding SaCas9 and VDR‐specific single‐guide RNA (sgRNA), and performed a series of behavioral experiments on these mice (**Figure** **2**A). Both the control and VDR‐knockdown (VDR‐KD) groups had similar body weights and showed comparable performance in the open field test, including total distance traveled, time spent in the center, and travel speed (Figure 2B–E, unpaired t‐test). These results suggest that neither the viral injection procedure nor VDR knockdown in the OB affects general motor function.

In the olfactory habituation test, both groups exhibited a significant decrease in sniffing time between the first and the fourth presentations of isoamyl acetate (Figure 2F, *P* < 0.0001 for control; *P* < 0.001 for VDR‐KD, one‐way repeated ANOVA with Tukey's multiple comparisons post hoc test) and a significant increase in sniffing time when the odor was switched to 2‐heptanone in the fifth trial (Figure 2F, *P* < 0.01 for control; *P* < 0.05 for VDR‐KD, one‐way repeated ANOVA with Tukey's multiple comparisons post hoc test). However, when presented with two highly similar odors (L)‐limonene and (D)‐limonene, the control group showed significantly increased in sniffing time during the fifth trial when the odor changed from (L)‐limonene to (D)‐limonene (Figure 2G, *P* < 0.0001, one‐way repeated ANOVA with Tukey's multiple comparisons post hoc test). In contrast, the VDR‐KD group displayed no change in sniffing time between the fourth and fifth trials (Figure 2G, one‐way repeated ANOVA with Tukey's multiple comparisons post hoc test), indicating impaired fine odor discrimination. Notably, both groups showed similar preference for (L) ‐ and (D)‐limonene, confirming that the mice had no intrinsic preference for either odor (Figure 2H, unpaired t‐test). Finally, the success of VDR knockdown was validated by quantitative western blot (Figure 2I, *P* < 0.0001, unpaired t‐test). Taken together, these findings suggest that reduced VDR expression in the OB impairs olfactory function in mice, highlighting the critical role of VitD signaling in maintaining olfactory circuit integrity and function.

### 2.3 Layer‐Specific and Cell‐Type‐Specific Expression of VDRs in the OB

**Figure 3:**
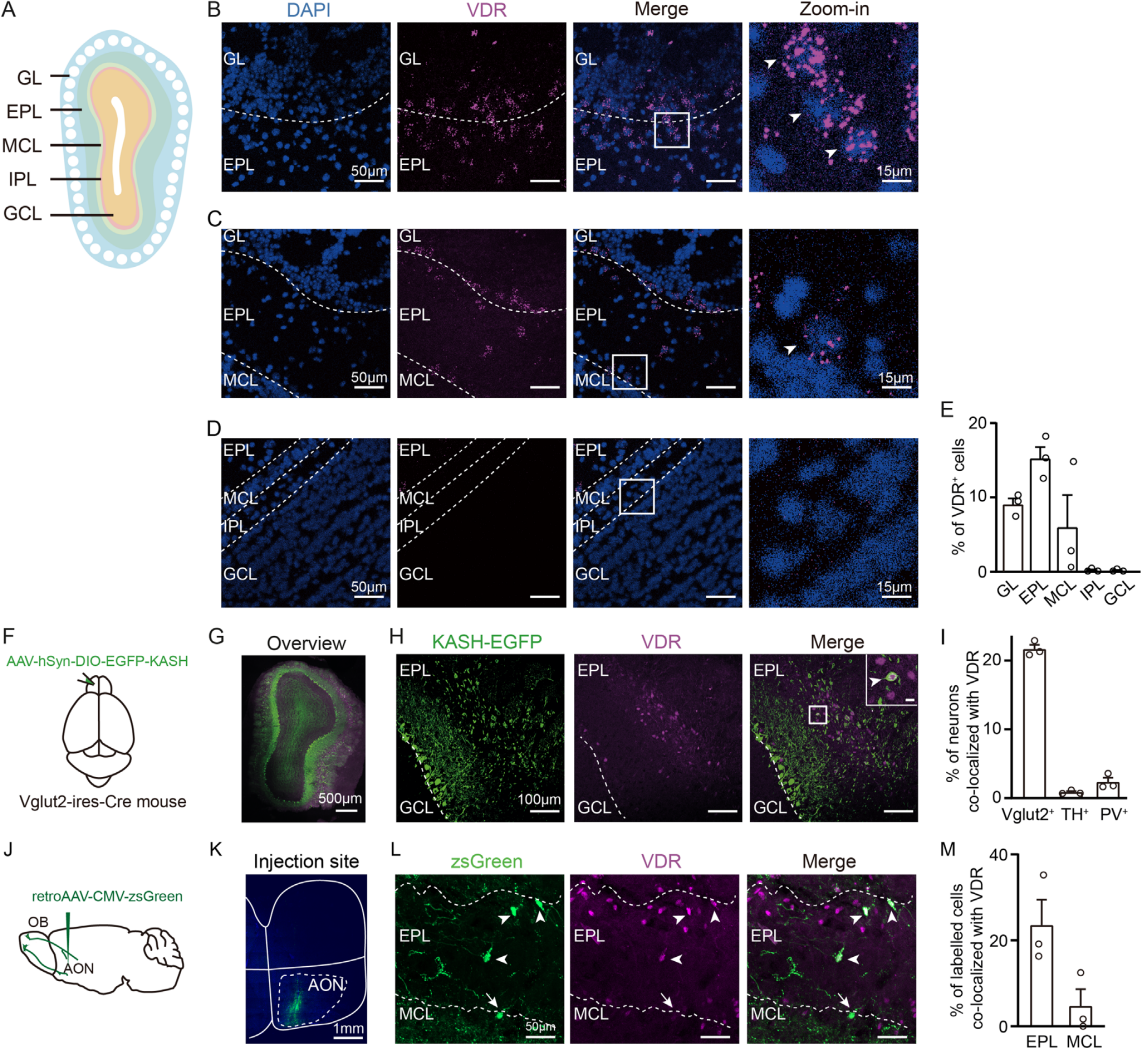
Layer‐specific and cell‐type‐specific expression of VDR in the mouse OB. A) Schematic of mouse OB layers (outermost to innermost): glomerular layer (GL), external plexiform layer (EPL), mitral cell layer (MCL), inner plexiform layer (IPL), and granule cell layer (GCL). B–D) Representative FISH images of adult mouse OB sections showing VDR mRNA signals (purple) and DAPI‐counterstained nuclei (blue). VDR expression depicted in GL/EPL (B), EPL/MCL (C), and IPL/GCL (D). Zoomed‐in images highlight the framed areas (white arrowheads indicate VDR^+^ cells). Scale bars: 50 µm (main), 15 µm (zoomed‐in). E) Quantification of VDR^+^ cells across OB layers. F) Schematic of experimental procedure. G) Panoramic view of OB section after viral infection: VDR (purple) and KASH‐EGFP (green). Scale bar: 500 µm. H) Immunohistochemistry of KASH‐EGFP (green) and VDR (purple) in EPL. Inset shows framed region. Scale bars: 100 µm (main), 10 µm (inset). I) Proportion of Vglut2^+^, TH^+^ and PV^+^ neurons co‐expressing VDR. J) Schematic of the experimental procedure. K) Anterior olfactory nucleus (AON) injection site overview. Scale bar: 1 mm. L) Immunohistochemistry of VDR (purple) in a section with retrograde‐labeled tufted cells (green, EPL) and mitral cells (green, MCL). White arrowheads: VDR^+^ tufted cells; arrows: VDR^–^ mitral cells. Scale bar: 50 µm. M) Percentage of retrogradely labeled tufted (EPL) and mitral (MCL) cells expressing VDR. Symbols = biological replicates; bars = mean ± SEM; *n* = 3 mice.

Given the demonstrated role of VitD levels in regulating olfactory function through VDRs in the OB, we next aimed to identify the specific OB cell populations expressing VDR. Using fluorescence in situ hybridization (FISH), we found VDR mRNA was most abundant in the external plexiform layer (EPL) and glomerular layer (GL), followed by the mitral cell layer (MCL), with minimal expression in the inner plexiform layer (IPL) and the granule cell layer (GCL) (**Figure** **3**A–E). VDR‐expressing (VDR^+^) cells in the EPL and GL accounted for 79.8% of the total VDR^+^ cells in the OB.

The EPL and GL contain excitatory tufted cells as well as inhibitory interneurons, such as periglomerular cells and short axon cells.^[^ ^33^, ^34^ ^]^ To comprehensively characterize VDR‐expressing cell types, we performed immunohistochemistry using neurotransmitter and molecular markers.^[^ ^35^, ^36^ ^]^ However, as VDR is a nuclear receptor while excitatory synaptic markers are primarily localized to synapses, assessing direct spatial relationships between VDR protein and these markers was technically challenging. To overcome this limitation, we stereotaxically injected AAV‐hSyn‐DIO‐EGFP ‐KASH into the OBs of *Vglut2‐ires‐cre* mice,^[^ ^37^ ^]^ enabling nuclear membrane‐tethered EGFP (via KASH) expression in excitatory *vGlut2* ^+^ neurons^[^ ^38^ ^]^ (Figure 3F,G). Our analysis revealed VDR expression in a subset (21.87%) of glutamatergic neurons within the EPL (Figure 3H,I). Since tufted cells and mitral cells represent the principal glutamatergic neuron populations in the OB, we next sought to determine which subtype preferentially expressed VDR. For this purpose, we injected retroAAV‐CMV‐zsGreen into the anterior olfactory nucleus (AON)—a known projection target of both tufted and mitral cells^[^ ^39^ ^]^—resulting in retrograde labeling of these neuronal populations (Figure 3J–L). Quantification demonstrated that 23.56% of EPL tufted cells expressed VDR, compared to only 4.72% of mitral cells in the MCL (Figure 3M).

Additionally, sparse co‐localization was observed between VDR^+^ cells in the EPL/GL and markers for inhibitory neurons—specifically, parvalbumin (PV; 2.82%) and tyrosine hydroxylase (TH; 0.85%), suggesting VDR expression in limited subsets of GABAergic and dopaminergic interneurons (Figure 3I; Figure S4A,B, Supporting Information). In contrast, VDR immunoreactivity did not overlap with calbindin (Calb1), calretinin (Calb2), somatostatin (SST), or S100b, indicating absence of detectable VDR expression in the majority of GABAergic interneuron subtypes and in glial cells (Figure S4C–F, Supporting Information). Collectively, these data demonstrate that VDRs are selectively expressed in excitatory tufted neurons and discrete subpopulations of inhibitory neurons within the EPL and GL.

### 2.4 VitD Signaling, Mediated by VDRs, Drives Cell‐Type‐Specific Transcriptomic Remodeling About the Synaptic Connectivity in Olfactory Neurons

**Figure 4:**
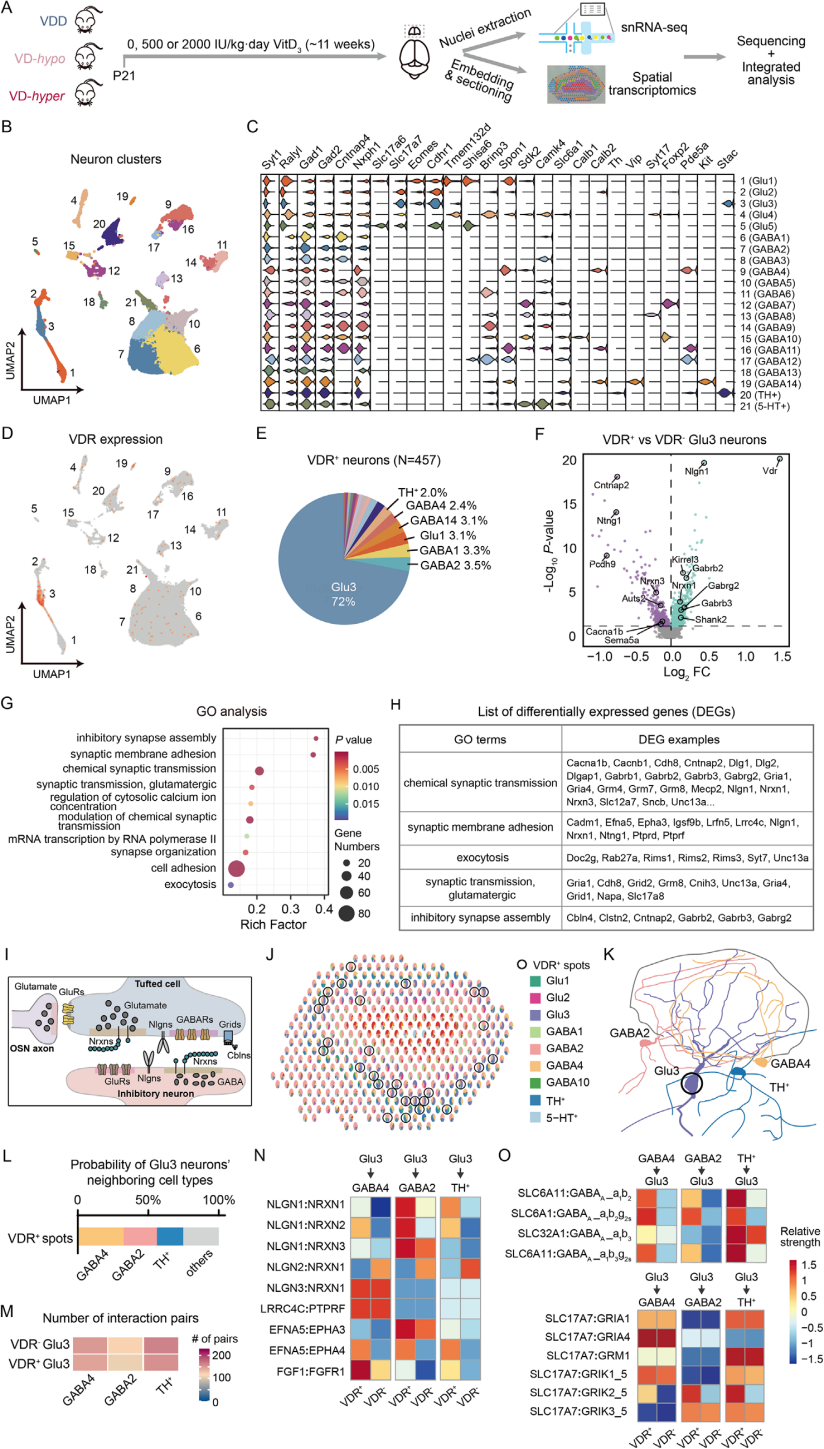
SnRNA‐seq and spatial transcriptomics reveal transcriptomic profiles and synaptic interaction properties of VDR^+^ neurons in the mouse OB. A) Schematic flowchart for snRNA‐seq (*n* = 2 mice per group) and spatial transcriptomic (*n* = 1 mouse per group) in OBs from mice fed diets containing three doses of VitD_3_. B) UMAP plot of neuronal clusters identified via snRNA‐seq. C) Violin plot showing expression levels of marker genes (columns) across 21 neuronal cell clusters (rows). D) UMAP plot depicting VDR^+^ neurons (red). E) Proportional distribution of VDR^+^ neurons across cell types (pie chart). F) Volcano plot of DEGs between VDR^+^ and VDR^−^ Glu3 neurons. *x*‐axis: log_2_ (fold change); *y*‐axis: −log_10_ (*P* value). G) GO annotation of DEGs between VDR^+^ and VDR^−^ Glu3 neurons. *x*‐axis: rich factor; *y*‐axis: GO terms; circle size: DEG count; color: *P* value. H) List of example DEGs in selected GO terms. I) Schematic of synaptic inputs to tufted cells: dendrodendritic synapses with GABAergic interneurons and excitatory synapses with OSNs. J) RCTD analysis showing representative spatial transcriptomics spots with cell‐type composition. Black circles denote VDR^+^ spots. K) Schematic of a Glu3 neuron and its predominant neighboring interneurons. L) Probability of finding GABA4, GABA2, or TH^+^ interneurons adjacent to VDR^+^ Glu3 neurons (RCTD spot co‐localization). M) Heatmap of interaction pairs between VDR^+^/VDR^−^ Glu3 neurons and neighboring interneurons (GABA4/GABA2/TH^+^). N) Synaptic adhesion molecule interaction heatmap comparing VDR^+^ and VDR^−^ Glu3 neurons with neighboring interneurons. O) Inhibitory (GABA_A_ receptors, top) and excitatory (bottom) synaptic interaction heatmaps comparing VDR^+^ and VDR^−^ Glu3 neurons with neighboring interneurons.

To unbiasedly identify the neuronal types in OB that respond to varying VitD levels and to investigate their cell‐type‐specific transcriptomic changes, we performed snRNA‐seq on OB tissues from six mice fed a gradient VitD diet (two mice per dietary group; **Figure** **4**A). We analyzed a total of 63739 nuclei from snRNA‐seq, with a median of 27997 unique molecular identifiers (UMIs) and 1514 genes detected per nucleus. These nuclei were annotated into 27 clusters based on established markers from the literature.^[^ ^36^, ^40^, ^41^ ^]^ These clusters included four excitatory neuron clusters (*Slc17a6* ^+^/*Slc17a7* ^+^) and 18 inhibitory neuron clusters (*Gad1* ^+^/*Gad2* ^+^). Additionally, we identified five clusters of glia cells, comprising three astrocyte clusters (*Aqp4^+^/Gldc^+^/Emid1^+^*), one microglia cluster (*Cx3cr1^+^/Hexb^+^*), and one oligodendrocyte cluster (*Mobp^+^/Plp1^+^*) (Figure S5A–C, Supporting Information). Notably, VDR expression was predominantly observed in neurons (99.35%), with only 0.65% of VDR^+^ cells identified as astrocytes (Figure S5D,E, Supporting Information), consistent with our immunohistochemistry findings.

Given these results, we focused on the 60501 nuclei classified as neurons and re‐analyzed them, identifying 21 clusters based on known subtype‐specific markers for excitatory projection neurons and GABAergic interneurons from previous studies^[^ ^36^, ^40^, ^41^ ^]^ (Figure 4B,C). Cluster 1‐5 represented glutamatergic neurons expressing *Slc17a6* (vGlut2) and/or *Slc17a7* (vGlut1). Among these, cluster 1‐3 express markers characteristic of mitral/tufted (M/T) neurons, including *Eomes* and *Cdhr1*,^[^ ^41^, ^42^ ^]^ while cluster 4 and 5 represented other excitatory neuron types in the OB. Cluster 6‐19 were GABAergic neurons marked by *Gad1^+^* and *Gad2^+^*. Specifically, cluster 9 (GABA4) and cluster 15 (GABA10) expressed interneuron markers (*Calb2^+^* and *Calb1^+^*, respectively) and represented two subtypes of periglomerular neurons in the GL.^[^ ^43^ ^]^ TH‐expressing cluster 20 (*TH^+^*), represented short axon neurons in the GL.^[^ ^43^ ^]^

We next sought to identify neuronal subtypes expressing VDR. Among the 457 VDR^+^ neurons analyzed, 72% (n = 329) belonged to cluster 3 (Glu3), a subpopulation of excitatory projection neurons expressing *vGlut1* (Figure 4D,E). These included 155 VDR^+^ Glu3 neurons from VDD, 103 from VD‐*hypo*, and 71 from VD‐*hyper*. The remaining VDR^+^ neurons included 3.5% from GABA2, 3.3% from GABA1 and 3.1% from Glu1 (Figure 4E). Notably, 18.45% of Glu3 neurons expressed VDRs, the highest proportion among all cell types, followed by 2.8% in GABA14 neurons and 0.63% in Glu1 neurons (Figure S5F and Table S1, Supporting Information). The cell‐type‐specific VDR expressions observed in the snRNA‐seq analysis aligns closely with our findings from FISH and immunohistochemistry. Thus, we identified excitatory Glu3 neurons exhibiting the highest VDR expression levels as predominantly tufted cells. These cells form dendrodendritic synapses with inhibitory interneurons, and, in some cases, receive excitatory input from olfactory sensory neurons (OSNs).^[^ ^44^, ^45^ ^]^

To explore cell‐type‐specific transcriptomic changes in the OB related to VitD, we first analyzed the Glu3 neurons and identified 1778 differentially expressed genes (DEGs) between VDR^+^ and VDR^−^ Glu3 neurons (*P* < 0.05, Table S2, Supporting Information). Notably, *VDR* itself exhibited the largest fold change between the two groups, validating our sequencing and analysis procedures (Figure 4F). Gene Ontology (GO) analysis of the DEGs revealed significant enrichment in pathways related to chemical synaptic transmission, synapse organizations and synaptic membrane adhesion (Figure 4G, *P* < 0.05, Table S3, Supporting Information). For example, genes associated with exocytosis, chemical synaptic transmission and synapse organization, such as *Rab27a*, *Unc13a*, *Cntnap2*, *Sncb*, *Dlg1*, *Dlg2* (*PSD93*), *Dlgap1*, glutamate receptor delta 1 (*Grid1)*, *Cbln4*, and *Shank2*, were differentially expressed between VDR^+^ and VDR^−^ Glu3 neurons. Additionally, genes encoding glutamate receptor (*Gria1*, *Gria4*, *Grm4*, *Grm7*, and *Grm8*) and GABA receptors (*Gabrb2*, *Gabrb3*, and *Gabrg2*) were differentially expressed. Genes associated with synaptic membrane adhesion, such as *Nlgn1*, *Nrxn1*, *Nrxn3*, *Ntng1*, *Lrrc4c*, *Epha3*, and *Efna5*, were also differentially expressed (Figure 4H). Given that VDR^+^ Glu3 neurons were predominantly identified as tufted cells, the differentially expressed transcripts encoded synaptic proteins critical for the reciprocal dendrodendritic synapses between tufted cells and GABAergic interneurons, as well as the excitatory postsynapses formed by OSNs (Figure 4I). These findings suggest that VDR‐mediated transcriptional regulation may play a pivotal role in modulating synaptic architecture within the OB, particularly in circuits involving tufted cells and their synaptic partners, which potentially shape sensory processing and integration in the OB.

We also examined VDR‐associated transcriptional differences in GABAergic neurons for comparison. Due to the small number of VDR^+^ GABAergic neurons (*n* = 91), they were analyzed collectively and compared to VDR^−^ GABAergic neurons (Figure S6A, *P* < 0.05, Table S4, Supporting Information). Interestingly, the pathways enriched in the DEGs between VDR^+^ and VDR^−^ GABAergic neurons shared some similarities but also exhibited distinct characteristics compared to Glu3 neurons. For instance, the synaptic adhesion molecule pathway (e.g., *Nlgns* and *Nrxns*), which was highly enriched in Glu3 neurons, was not significant in GABAergic neurons. Moreover, while pathways related to chemical synaptic transmission and synapse organization were enriched, the specific genes showing differential expression were distinct (Figure S6B,C and Table S5, Supporting Information). For example, genes such as *Lrp4* and *Slc9a6* are differentially expressed in GABAergic neurons based on VDR expression but not in the Glu3 neurons. These findings underscore the cell‐type‐specific nature of VDR‐mediated transcriptional regulation, and highlight the intricate and context‐dependent role of VDR signaling in shaping the synaptic and functional properties of distinct neuronal populations.

We next aimed to investigate whether the neuronal interactions of Glu3 neurons, mediated by specific pairs of signaling molecules, were influenced by VDR expression. To address this, we first identified the neuronal populations spatially associated with VDR^+^ Glu3 neurons using a combination of 10X spatial transcriptomics and our snRNA‐seq data (Figure 4A). The OB from one mouse per VitD dietary group (VDD, VD‐*hypo*, and VD‐*hyper*) was sequenced, yielding a total of 5346 spots (55 µm per spot) in the spatial transcriptomics assay. This analysis revealed 11 distinct clusters corresponding to the six layers of the OB (Figure S7A–D, Supporting Information). Among these, 367 spots were identified as VDR^+^ (*n* = 107 for VDD, *n* = 61 for VD‐*hypo*, *n* = 199 for VD‐*hyper*). VDR^+^ spots were predominantly classified as cluster 7 (40.3%), cluster 1 (18.8%), and cluster 2 (14.2%), which primarily correspond to the GL and EPL, corroborating our FISH data (Figure S7E, Supporting Information).

To infer the predominant neuronal types within each spot, we employed robust cell type decomposition (RCTD) based on transcriptional profiles of major neuronal populations (defined as those with ≥ 1000 cells) derived from snRNA‐seq data.^[^ ^46^ ^]^ This approach ensured robust transcripts representation for accurate cell type identification (Figure 4J; Figure S7G–V, Supporting Information). For each spot, the top four cell types identified by RCTD were included in subsequent analyses. Approximately 80% of VDR^+^ spots across all dietary groups contained Glu3 neurons, consistent with our earlier snRNA‐seq findings (Figure S7F, Supporting Information).

We then focused on the VDR^+^ spots when Glu3 neurons were identified as the primary cell type by RCTD (*n* = 177 total). Analysis of these Glu3‐containing VDR^+^ spots revealed that GABA4, GABA2, and TH^+^ neurons were the most frequently observed neighboring cell types (Figure 4K,L). Notably, GABA4 neurons, characterized by *Calb2^+^* expression, represent a subtype of periglomerular neurons, while *TH^+^* neurons are short axon neurons. Both are inhibitory interneuron types typically localized near tufted cells in the GL and outer EPL. Thus, the integration of snRNA‐seq and spatial transcriptomics successfully delineated the interneuron population adjacent to Glu3 neurons.

To further explore neuronal interactions, we next employed Cellphone DB v5,^[^ ^47^ ^]^ a computational tool that predicts cell‐cell communication by analyzing ligand‐receptor interactions in our snRNA‐seq data. We focused on interactions between VDR^+^ or VDR^−^ Glu3 neurons and neighboring GABA4, GABA2, or TH^+^ interneurons (Table S6, Supporting Information). While the overall number of interaction pairs between Glu3 neurons and these interneurons was similar regardless of VDR expressing (Figure 4M), interactions involving synaptic adhesion molecules—such as Nlgn1 with three Nrxn isoforms—were predicated to be stronger in VDR^+^ Glu3 neurons than in VDR^−^ Glu3 neurons (Figure 4N). Similarly, interaction involving Efna5‐Epha3/4 and FGF1‐FGFR1 were possibly more pronounced in VDR^+^ Glu3 neurons. In contrast, interactions involving Nlgn3‐Nrxn1 and LRRC4C‐PTPRF showed comparable strength irrespective of VDR status (Figure 4N).

Furthermore, inhibitory synaptic interactions mediated by GABA_A_ receptors subunits (e.g., a1, b2, b3, and g2) were likely stronger in VDR^+^ Glu3 neurons than in VDR^−^ neurons (Figure 4O, upper panel). Conversely, most excitatory synaptic interactions involving glutamate receptors—such as AMPA receptor subunits (Gria1 and Gria4), metabotropic glutamate receptor 1 (GRM1 or mGluR1), kainate receptor subunit (e.g., Grik1/5 and Grik3/5)—exhibited similar strength regardless of VDR expression. However, the interaction involving kainate receptor subunit Grik2/5 was potentially stronger in VDR^+^ Glu3 neurons compared to VDR^−^ Glu3 neurons (Figure 4O, lower panel).

In summary, the integration of Cellphone DB analysis with snRNA‐seq and spatial transcriptomics data predicted that VDR^+^ Glu3 neurons—primarily tufted cells—probably engage in more robust and nuanced interactions with neighboring periglomerular and short axon cells within the glomerular layer. These interactions are potentially enriched for synaptic adhesion molecules, including Nlgn‐Nrxn pairs, as well as specific subtypes of glutamate and GABA_A_ receptors, which collectively suggest VDR‐mediated VitD signaling may refine synaptic connectivity in this neuronal population.

### 2.5 VitD Modulates Synaptic Pathways in the OB via VDR‐Dependent Mechanisms

To investigate the dose‐dependent effects of VitD mediated by VDRs, we focused on VDR^+^ Glu3 neurons from snRNA‐seq and Glu3‐containing VDR^+^ spots within anatomically defined layers (corresponding to clusters 1, 2, and 7 in the GL and outer EPL) from the spatial transcriptomics.

**Figure 5:**
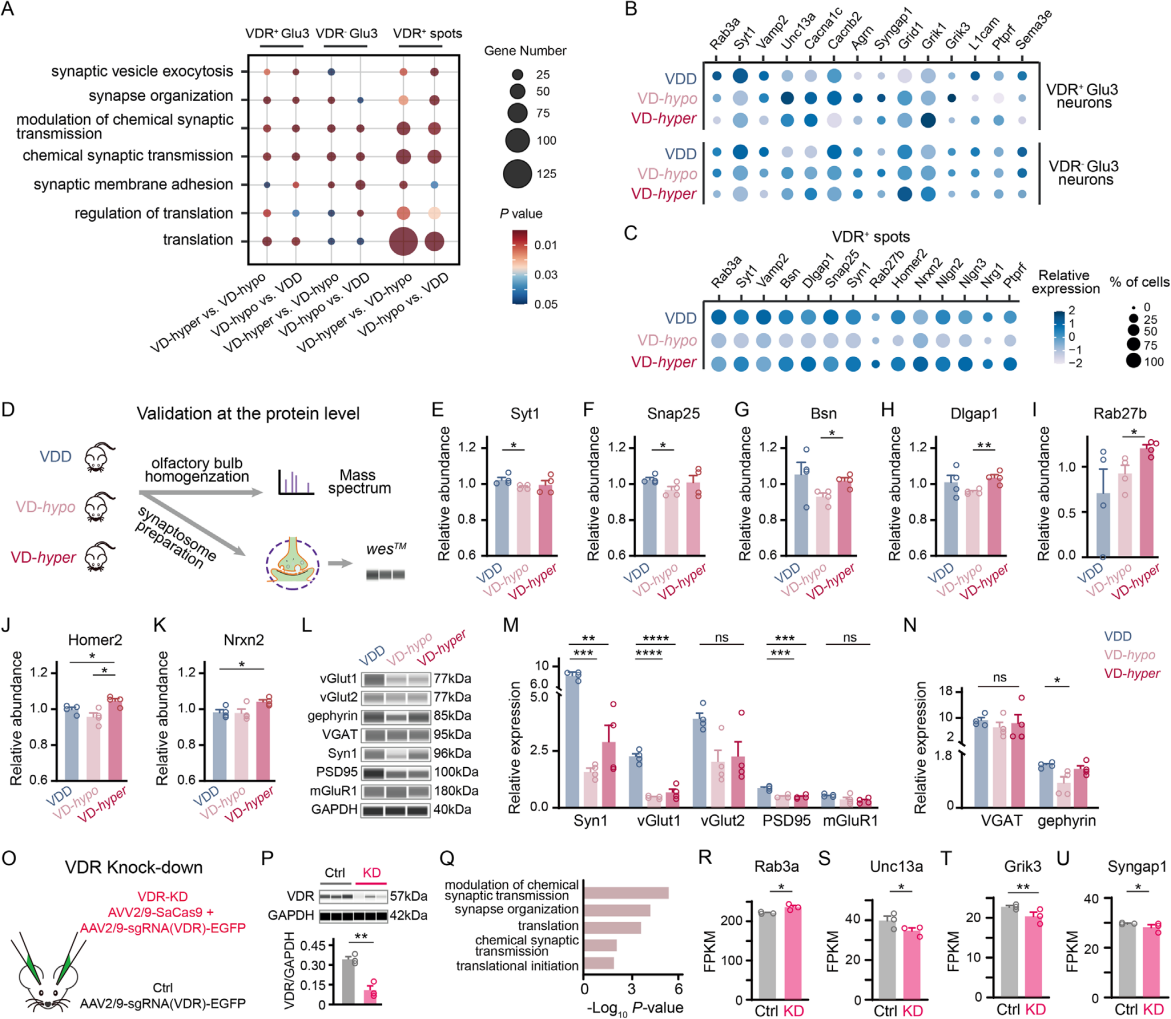
VitD modulates synaptic pathways in the OB via VDR‐dependent mechanisms. A) GO annotation of DEGs between VD‐*hyper* versus VD‐*hypo* and VD‐*hypo* versus VDD in VDR⁺ and VDR^−^ Glu3 neurons from snRNA‐seq (*n *= 2 mice per group) and VDR⁺ spots from spatial transcriptomics (*n* = 1 mouse per group). *x*‐axis: comparison groups; *y*‐axis: GO terms; circle size: DEG count; color: *P* value. B,C) Dot plots of synaptic function‐related DEGs in VDR⁺/VDR^−^ Glu3 neurons (snRNA‐seq, B) and VDR⁺ spots (spatial transcriptomics, C) across VD‐*hyper*, VD‐*hypo*, and VDD groups. Dot size: percentage of expressing cells; color: mean scaled expression. D) Experimental schematic of protein‐level validations: LC‐MS/MS‐based proteomic analysis of whole OB lysates and synaptosome plus quantitative western blot (*wes*) analysis (VDD, VD‐*hypo*, and VD‐*hyper*). E–K) Relative expression levels of Syt1 (E), Snap25 (F), Bsn (G), Dlgap1 (H), Rab27b (I), Homer2 (J), and Nrxn2 (K) in LC‐MS/MS‐based proteomic analysis (*n* = 4 mice per group; unpaired t‐test). L–N) Synaptosomal excitatory (L, M) and inhibitory (L, N) synaptic protein levels (L: representative *wes* image; M and N: corresponding quantifications; *n* = 4 mice per group; one‐way ANOVA with Tukey's multiple comparisons). O) Experimental schematic of SaCas9‐mediated VDR‐KD in the mouse OB. P) The *wes* image and quantification of VDR in VDR‐KD and control (Ctrl) OB (*n* = 3 mice per group). Q) GO annotation of VDR‐KD versus Ctrl DEGs (*n* = 3 mice per group). *x*‐axis: −log_10_ (*P* value); *y*‐axis: GO terms. R–U) Relative mRNA expression of *Rab3a* (R), *Unc13a* (S), *Grik3* (T), *Syngap1* (U) (VDR‐KD versus Ctrl; bulk RNA‐seq; *n* = 3 mice per group). Data normalized and corrected for multiple testing. Symbols = biological replicates; bars = mean ± SEM. Significance levels: *P < 0.05, **P < 0.01, ***P < 0.001, ****P < 0.0001; ns: not significant. FPKM: Fragments per kilobase million.

GO analysis revealed that both VDR^+^ Glu3 neurons and Glu3‐containing VDR^+^ spots were significantly enriched in translational regulation pathways in a VitD dose‐dependent manner, whereas this enrichment was absent in VDR^−^ Glu3 neurons (**Figure** **5**A, *P* < 0.05, Table S7, Supporting Information), suggesting that VitD specifically modulates translation in VDR‐expressing Glu3 neurons, potentially contributing to functional changes in the OB. In contrast, both VDR^+^ and VDR^−^ Glu3 neurons, as well as Glu3‐containing VDR^+^ spots, were significantly enriched in pathways associated with synaptic organization and chemical synaptic transmission under varying dietary VitD intake (Figure 5A), indicating synaptic pathways might be influenced by both VDR‐dependent and ‐independent mechanisms.

To identify synaptic effects specifically mediated by VDR, we analyzed DEGs associated with synaptic functions that were altered in VDR^+^ Glu3 neurons, but not in VDR^−^ Glu3 neurons, under varying VitD levels (*P* < 0.05,〡Log_2_FC〡≥ 0.15, Table S8, Supporting Information). Among presynaptic molecules, transcripts encoding *Rab3a*, *Syt1*, and *Vamp2* were significantly upregulated in VDR^+^ Glu3 neurons from the VDD group compared to supplemented groups (Figure 5B). Conversely, *Unc13a*, voltage‐gated calcium channel *Cacna1c*, and *Cacnb2* were most highly expressed in VDR^+^ Glu3 neurons from the VD‐*hypo* groups (Figure 5B). Among postsynaptic molecules, transcripts for *Agrn* and *Syngap1* exhibited the lowest expression in VDR^+^ Glu3 neurons under VitD deficiency (Figure 5B). Regarding glutamate receptor transcripts, *Grik1* and *Grid1* showed minimal expression in the VDD group, whereas *Grik3* was most highly expressed in the VD‐*hypo* group (Figure 5B). Additionally, synaptic membrane adhesion molecules, including *L1cam*, *Ptprf*, and *Sema3e*, displayed altered expression in VDR^+^ Glu3 neurons depending on serum VitD levels (Figure 5B). Critically, none of these changes were observed in VDR^−^ Glu3 neurons (Figure 5B), indicating that VDR mediates VitD dose‐dependent regulation of specific synaptic molecule expression.

A parallel analysis of VDR^+^ spots revealed that multiple synaptic molecules—including *Rab3a*, *Syt1*, *Vamp2*, *Bsn*, *Dlgap1*, *Snap25*, *Syn1*, and *Homer2* —as well as synaptic adhesion molecules such as *Nlgn2*, *Nlgn3*, *Nrg1*, and *Ptprf*, exhibited higher expression in the VDD and VD‐*hyper* groups compared to the VD‐*hypo* group (Figure 5C, *P* < 0.05; and Table S9, Supporting Information). In contrast, *Rab27b* and *Nrxn2* were least expressed in the VDD group and most highly expressed in the VD‐*hyper* group (Figure 5C). In summary, these findings establish that differential VitD levels modulate synaptic protein expression specifically in VDR‐expressing Glu3 neurons and discrete VDR‐positive spots, revealing a cell‐type‐specific molecular mechanism through which dietary VitD intake regulates olfactory synapses.

Furthermore, we analyzed DEGs in VDR^+^ GABAergic neurons under varying VitD conditions (*P* < 0.05, Table S10, Supporting Information). Similar to Glu3 neurons, translation emerged as one of the top enriched pathways in GABAergic neurons. However, pathways related to synapse organization and chemical synaptic transmission were less prominent in GABAergic neurons, indicating a degree of cell‐type‐specific regulation in VitD signaling (Figure S6D–F and Table S11, Supporting Information). Collectively, these findings underscore shared patterns across spatial and single‐nucleus transcriptomic datasets, highlighting translation and synaptic functions as key physiological domains significantly modulate by VitD levels.

Although the snRNA‐seq and spatial transcriptomic analyses were performed with cell‐type or spot‐type specificity, bulk RNA sequencing of the OB from VDD, VD‐*hypo* and VD‐*hyper* mice further corroborated that molecules associated with synaptic functions and translations were strongly influenced by serum VitD levels (Figure S8A,B and Table S12, Supporting Information). Gene set enrichment analysis (GSEA) of DEGs between the VD‐*hyper* and VD‐*hypo* groups, as well as the VD‐*hyper* and VDD groups, revealed significant enrichment in pathways such as regulation of neurotransmitter receptor activity, maintenance of synapses structures, olfactory transduction, ribosome function (Figure S8C,D, Supporting Information, *P* < 0.05). Moreover, GO analysis of the DEGs between these two comparisons revealed significant enrichment in pathways such as chemical synaptic transmissions, cytoplasmic translation, and negative regulation of target of rapamycin complex1 (TORC1) signaling—a pathway implicated in translation regulation (Figure S8E,F, *P* < 0.05, Table S13, Supporting Information). These results demonstrate that varying levels VitD intake exert significant effects on synaptic functions and translational processes within the OBs, consistent with the snRNA‐seq and spatial transcriptomics results.

To validate the VitD‐dependent changes in synaptic molecules at the protein level, we employed two complementary approaches (Figure 5D). First, we conducted quantitative proteomic analysis using liquid chromatography‐tandem mass spectrometry (LC‐MS/MS) to identify differentially expressed proteins (DEPs) in the OB of VDD, VD‐*hypo* and VD‐*hyper* groups (Table S14, Supporting Information). Synaptic proteins such as Syt1 and Snap25 were significant upregulated in the VDD group compared to the VD‐*hypo* group (Figure 5E,F, *P* < 0.05, unpaired t‐test), while Bsn and Dlgap1 were significantly downregulated in the VD‐*hypo* group compared to VD‐*hyper* group (Figure 5G,H, *P* < 0.05 for Bsn, *P* < 0.01 for Dlgap1, unpaired t‐test). Additionally, Rab27b, Homer2, and Nrxn2 exhibited the highest expression levels in the VD‐*hyper* group (Figure 5I–K, *P* < 0.05, unpaired t‐test).

Second, we performed quantitative western blot (*wes*) on synaptic proteins in the synaptosome isolated from the OB of mice fed with gradient VitD diets. Excitatory presynaptic proteins, including Syn1 and vGlut1, as well as the postsynaptic protein PSD95, were significantly upregulated in the VDD group compared to the VitD‐supplemented groups (Figure 5L,M, Syn1: *P *< 0.001 versus VD‐*hypo*, *P* < 0.01 versus VD‐*hyper*; vGlut1: *P* < 0.0001; PSD95: *P* < 0.001; one‐way ANOVA with Tukey's post hoc test). Similarly, vGlut2 and mGluR1 showed a trend toward increased expression in the VDD group compared to the VD‐*hypo* and VD‐*hyper* groups, although these changes did not reach statistical significance (Figure 5L,M, one‐way ANOVA with Tukey's multiple comparisons post hoc test). Furthermore, the inhibitory postsynaptic protein gephyrin was significantly upregulated in the VDD group compared to the VD‐hypo group (Figure 5L,N, *P* < 0.05, one‐way ANOVA with Tukey's post hoc test). In contrast, the inhibitory presynaptic proteins VGAT remained unchanged across different VitD levels (Figure 5L,N, one‐way ANOVA with Tukey's multiple comparisons post hoc test).

In summary, the protein‐level findings for synaptic molecules were consistent with the results from snRNA‐seq and spatial transcriptomics. Together, these results demonstrate a pronounced upregulation of excitatory synaptic proteins and a moderate upregulation of inhibitory synaptic proteins in the OB under VitD deficiency. These molecular changes may provide a potential mechanism underlying behavioral alterations in olfactory function associated with varying VitD intake.

We next sought to determine whether the observed changes in synaptic protein expression in the OB under gradient serum VitD levels were mediated by the VDR. To investigate this, we knocked down VDR expression in the OB using a viral‐mediated SaCas9 approach (Figure 5O). This achieved a reduction of VDR protein levels by over 60% in the KD group compared to controls (Figure 5P, *P* < 0.01, unpaired t‐test). Bulk RNA sequencing followed by GO analysis of DEGs revealed significant enrichment in pathways related to synaptic functions and translation, consistent with our earlier results (Figure 5Q, *P* < 0.05; Tables S15 and S16, Supporting Information). Notably, transcripts encoding synaptic molecules that were specifically modulated in VDR^+^ Glu3 neurons under varying VitD levels—*Rab3a*, *Unc13a*, *Grik3*, and *Syngap1*—showed significant changes in the KD group (Figure 5R–U, *P* < 0.05 for *Rab3a*, *Unc13a*, and *Syngap1*; *P* < 0.01 for Grik3). Furthermore, presynaptic molecules, including *Snca* and *Slc7a11*, were significantly upregulated in the KD group (Figure S9A,B, Supporting Information, *P* < 0.05 for both), whereas the presynaptic molecules *Bsn* and *Cacna1a*, as well as postsynaptic molecules *mGluR1* and *Shank1*, were significantly downregulated (Figure S9C‐F, Supporting Information, *P* < 0.01 for *Bsn* and *Cacna1a*, *P* < 0.05 for *mGluR1*, *P* < 0.0001 for *Shank1*). Additionally, synaptic adhesion molecules, such as *Nlgn3* and *Nrg1*, exhibited significant changes in the KD group (Figure S9G,H, Supporting Information, *P* < 0.05 for both). These results closely aligned with our sequencing and protein‐level analyses, further underscoring the critical role of VDR in regulating synaptic protein expression in the OB.

### 2.6 VitD Regulates Translation—Particularly through Mechanistic Target of Rapamycin (mTOR) Signaling—in Olfactory Neurons and Synapses via VDR‐Dependent Transcriptional Control

**Figure 6:**
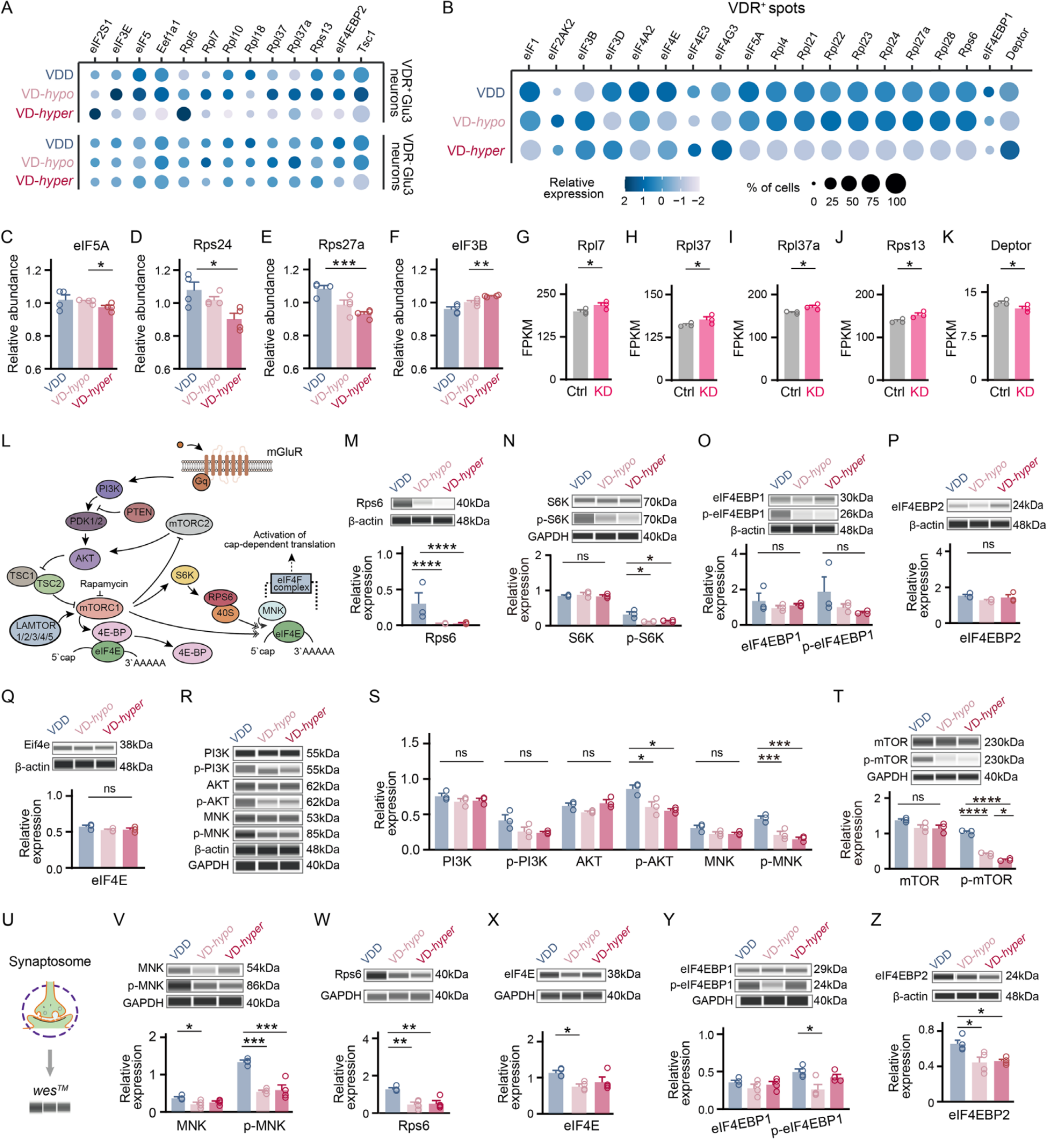
VitD regulates translation—particularly through mTOR signaling—in olfactory neurons and synapses via VDR‐dependent transcriptional control. A,B) Dot plots of translation‐related DEGs in VDR⁺/VDR^−^ Glu3 neurons (snRNA‐seq, A) and VDR⁺ spots (spatial transcriptomics, B) across VD‐*hyper*, VD‐*hypo*, and VDD groups. Dot size: percentage of expressing cells; color: mean scaled expression. C–F) Relative expression levels of eIF5A (C), Rps24 (D), Rps27a (E), and eIF3B (F) in LC‐MS/MS‐based proteomic analysis (*n* = 4 mice/group; unpaired t‐test). G–K) Relative mRNA expression of *Rpl7* (G), *Rpl37* (H), *Rpl37a* (I), *Rps13* (J), and *Deptor* (K) (VDR‐KD versus Ctrl; bulk RNA‐seq; *n* = 3 mice per group). Data normalized and corrected for multiple testing. L) Schematic of mTOR/translation signaling pathway. M–Q) *Wes* quantification of mTOR signaling components in OB lysates: Rps6 (M), S6K and p‐S6K (N), eIF4EBP1 and p‐eIF4EBP1 (O), eIF4EBP2 (P), and eIF4E (Q) (*n* = 3 mice per group; upper panels: representative *wes* images; lower panels: corresponding quantifications; one‐way ANOVA with Tukey's multiple comparisons). R–T) *Wes* quantification of mTOR/translation regulators (R‐S), mTOR/p‐mTOR (T) in OB lysates (*n* = 3 mice per group; upper panels: representative *wes* images; lower panels: corresponding quantifications; one‐way ANOVA with Tukey's multiple comparisons). U) Workflow for synaptosome isolation and proteomic validation. V–Z) Synaptosomal mTOR/translation machinery components: MNK and p‐MNK (V), Rps6 (W), eIF4E (X), eIF4EBP1 and p‐eIF4EBP1 (Y), and eIF4EBP2 (Z) (*n* = 4 mice per group; upper panels: representative *wes* images; lower panels: corresponding quantifications; one‐way ANOVA with Tukey's multiple comparisons). Symbols = biological replicates; bars = mean ± SEM. Significance levels: *P < 0.05, **P < 0.01, ***P < 0.001, ****P < 0.0001; ns: not significant. FPKM: Fragments per kilobase million.

Given our snRNA‐seq, spatial transcriptomics, bulk RNA sequencing and VDR knockdown analyses collectively indicated a strong regulation on translation by VitD levels, we next investigated the expression of translation‐related genes in VDR^+^ Glu3 neurons and VDR^+^ spots under varying VitD conditions. DEGs encoding translation initiation factors—such as *eIF3E* and *eIF5*—as well as the elongation factor *eEF1A1* and ribosome proteins (*Rpl10*, *Rpl18*, and *Rps13*), exhibited the lowest expression in VDR^+^ Glu3 neurons from the VD‐*hyper* group (**Figure** **6**A, *P* < 0.05, 〡Log_2_FC〡≥ 0.15; and Table S8, Supporting Information). In contrast, *eIF2S1* and *Rpl5* showed the highest expression in the VD‐*hyper* group, while *Rpl7*, *Rpl37* and *Rpl37a* were most highly expressed in the VD‐*hypo* group (*P* < 0.05, 〡Log_2_FC〡≥ 0.15, Table S8, Supporting Information). Notably, these changes were not detected in VDR^−^ Glu3 neurons, suggesting that translational regulation exerted by VitD depends on VDR expression in a given cell type (Figure 6A).

Interestingly, many translation initiation factors—including *eIF1*, *eIF3D*, *eIF4A2*, *eIF4E*, and *eIF5A*—were most highly expressed in VDR^+^ Glu3‐containing spots from the VDD group, whereas most differentially expressed ribosomal proteins (*Rpl4*, *Rpl21*, *Rpl22*, *Rpl23*, *Rpl24*, *Rpl27a*, and *Rpl28*) showed the lowest expression in VDR^+^ Glu3‐containing spots from the VD‐*hyper* group (Figure 6B, *P* < 0.05; and Table S9, Supporting Information). These results suggest that VDD is associated with elevated translation activity, while high‐dose VitD supplementation suppresses translation levels. Notably, molecules associated with mTOR signaling—*TSC1*, eIF4E binding protein 1 (*eIF4EBP1*), eIF4E binding protein 2 (*eIF4EBP2*), *Deptor* and *Rps6*—also displayed differential expression in VDR^+^ Glu3 neurons or VDR^+^ Glu3‐containing spots upon varying serum VitD levels, suggesting mTOR signaling is involved in VDR‐mediated translational regulation (Figure 6A,B).

Quantitative proteomic analysis of DEPs in the OB of VDD, VD‐*hypo* and VD‐*hyper* groups using LC‐MS/MS revealed that eIF5A, Rps24, and Rps27a exhibited the lowest expression in the VD‐*hyper* group (Figure 6C–E; and Table S14, Supporting Information, *P* < 0.05 for eIF5A and Rps24, *P* < 0.001 for Rps27a, unpaired t‐test). In contrast, eIF3B was significantly upregulated in the VD‐*hyper* group (Figure 6F, *P* < 0.01, unpaired t‐test). These findings were consistent with results from snRNA‐seq and spatial transcriptomics.

Notably, viral‐mediated knockdown of VDR in the OB using the SaCas9 system led to a significant upregulation of transcripts encoding ribosomal proteins, such as *Rpl4*, *Rpl7*, *Rpl21*, *Rpl37*, *Rpl37a*, and *Rps13* (Figure 6G–J; Figure S9I,J, Supporting Information, *P* < 0.05). Interestingly, *Deptor*, a component of the mTOR complex, was significantly decreased in the OBs of VDR‐KD mice (Figure 6K, *P* < 0.05). These findings underscore the critical role of VDR in modulating translation‐related processes in a VitD dose‐dependent manner, linking its regulatory function to the mTOR signaling pathway.

Given the differential expression of mTOR signaling components (*eIF4EBP1*, *eIF4EBP2*, *eIF4E*, and *Rps6*) under varying VitD levels (Figure 6L), we performed quantitative western blot of these proteins in the OBs of VDD, VD‐*hypo*, and VD‐*hyper* mice. Rps6, as well as phosphorylated ribosomal protein S6 kinase (p‐S6K), a downstream effector of mTOR signaling that directly regulates translation, were significantly upregulated in the VDD group compared the VD*‐hypo* and VD‐*hyper* groups (Figure 6M,N; *P* < 0.0001 for Rps6 and *P* < 0.05 for p‐S6K; one‐way ANOVA with Tukey's multiple comparisons post hoc test). These findings are consistent with enhanced translation activity during VitD deficiency, likely associated with hyperactivation of mTOR signaling. Moreover, S6K, eIF4EBP1, phosphorylated eIF4EBP1 (p‐eIF4EBP1), eIF4EBP2 and eIF4E showed no differential expression across the three groups (Figure 6N–Q, one‐way ANOVA with Tukey's multiple comparisons post hoc test).

To explore the upstream mechanisms driving mTOR signaling and translational activation in the VDD group, we investigated known regulators of mTOR and translation^[^ ^48^ ^]^ (Figure 6L). The VDD group showed significantly elevated levels of phosphorylated AKT serine/threonine kinase (p‐AKT) and phosphorylated mTOR (p‐mTOR) compared to VitD‐supplemented groups (Figure 6R–T, and p‐AKT: *P* < 0.05; p‐mTOR: *P* < 0.0001; one‐way ANOVA with Tukey's multiple comparisons post hoc test). In contrast, total protein levels of PI3K, phosphorylated PI3K (p‐PI3K), AKT and mTOR remained unchanged across all groups (Figure 6R–T, one‐way ANOVA with Tukey's multiple comparisons post hoc test), suggesting that mTOR signaling activation under VitD deficiency specifically involves AKT pathway activation. Regarding translational regulators, while mitogen‐activated protein kinase‐interacting kinase (MNK) expression was similar among groups, phosphorylated MNK (p‐MNK) was significantly increase in the VDD group (Figure 6R,S and p‐MNK: *P* < 0.001). Collectively, these findings not only validated the earlier sequencing data but also demonstrated that increased translation during VitD deficiency is, at least in part, mediated by mTOR signaling activation.

We next investigated whether the observed upregulation of translation during VitD deficiency also occurs at the synaptic level. Quantitative analysis of synaptosome proteins revealed a significant increase in MNK, p‐MNK, Rps6, eIF4E, and p‐eIF4EBP1 in the VDD group (Figure 6U–Y, MNK: *P* < 0.05 versus VD‐*hyper*; p‐MNK: *P* < 0.001; Rps6: *P* < 0.01; eIF4E: *P* < 0.05 versus VD‐*hypo*; p‐eIF4EBP1: *P* < 0.05 versus VD‐*hypo*; one‐way ANOVA with Tukey's multiple comparisons post hoc test), suggesting an increased mTOR signaling and translation at the synapse level. Moreover, eIF4EBP2 expression was significantly elevated in the VDD group compared to the VitD‐supplemented groups (Figure 6Z, *P* < 0.05, one‐way ANOVA with Tukey's multiple comparisons post hoc test). These results indicate that mTOR signaling drives an upregulation of local translation at synapses during VitD deficiency, which may directly contribute to changes in synaptic protein composition and functions.

### 2.7 mTOR Inhibition Rescues Olfactory Deficits in VitD‐Deficient Mice by Normalizing Translation and Excitatory Synaptic Protein Expression

**Figure 7:**
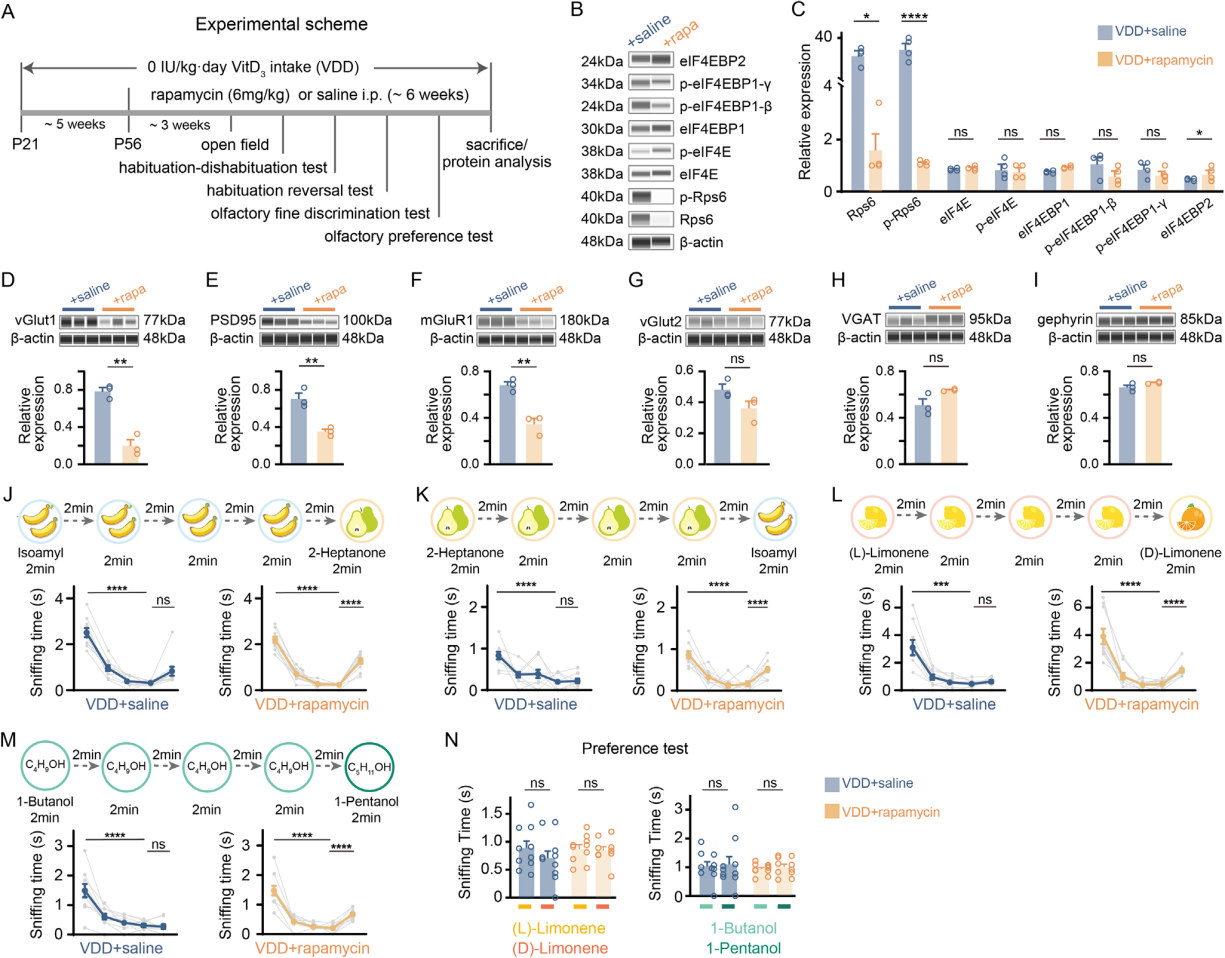
mTOR inhibition rescues olfactory deficits in VitD‐deficient mice by normalizing translation and excitatory synaptic protein expression. A) Experimental scheme of behavioral tests conducted on rapamycin‐treated (rapa) and control (saline) groups. VDD mice received intraperitoneal rapamycin (6 mg kg^−1^) or saline 3 times/week from postnatal day 56 (P56) for 6 weeks, with behavioral tests beginning at 3 weeks post‐treatment. B,C) *Wes* quantification of mTOR‐translation pathway components in OB lysates (*n* = 4 mice per group; B: representative *wes* images; C: corresponding quantifications; Mann‐Whitney U test for Rps6; unpaired t‐test for other proteins). D–I) Synaptosomal synaptic protein levels: vGlut1 (D), PSD95 (E), mGluR1 (F), vGlut2 (G), VGAT (H), and gephyrin (I) (*n* = 3 mice per group; upper panels: representative *wes* images; lower panels: corresponding quantifications; unpaired t‐test), with vGlut1, PSD95, and vGlut2 (D, E, G) normalized to a common control β‐actin; VGAT and gephyrin (H, I) to their common control β‐actin; and mGluR1 (F) to its own control β‐actin. J–M) Sniffing time in the olfactory habituation‐dishabituation test (J, L, M) and olfactory habituation reversal test (K) (*n* = 10 mice per group; one‐way repeated ANOVA with Tukey's multiple comparisons). Odor pairs: isoamyl acetate versus 2‐heptanone (J, K); (L)‐limonene versus (D)‐limonene (L); 1‐butanol versus 1‐pentanol (M). N) Sniffing time for (L)‐limonene versus (D)‐limonene (left) and 1‐butanol versus 1‐pentanol (right) in the olfactory preference test (*n* = 10 mice per group; unpaired t‐test). Symbols = biological replicates; bars = mean ± SEM. Significance levels: *P < 0.05, **P < 0.01, ***P < 0.001, ****P < 0.0001; ns: not significant.

Given that VDD mice exhibited impaired olfactory function alongside increased translation and synaptic protein expression, and considering the association of the mTOR signaling pathway with the VitD‐dependent translational regulation, we next investigated whether suppressing overactivated translation through mTOR inhibition could rescue synaptic and behavioral deficits caused by VitD deficiency. To this end, we treated VDD mice with rapamycin, a specific mTOR inhibitor, and conducted a series of experiments (**Figure** **7**A).

First, rapamycin treatment significantly reduced the protein levels of Rps6 and slightly increased eIF4EBP2 levels in the OB of VDD mice, without altering the expression of eIF4E, eIF4EBP1, or p‐EIF4EBP1 (Figure 7B,C, *P* < 0.05 for Rps6, Mann‐Whitney U test; *p* < 0.0001 for p‐Rps6 and *P* < 0.05 for eIF4EBP2, unpaired t‐test). These results indicate that rapamycin effectively downregulates mTOR‐associated translational machinery. Furthermore, in synaptosome fractions, the expression of excitatory synaptic markers—vGlut1, mGluR1, and PSD95—was significantly reduced following rapamycin treatment (Figure 7D–F, *P* < 0.01, unpaired t‐test), while vGlut2 and markers of inhibitory synapse (VGAT and gephyrin) remained unchanged (Figure 7G–I, unpaired t‐test). These findings suggest that mTOR‐dependent translation plays a specific role in modulating the expression of excitatory synaptic proteins under conditions of VitD deficiency.

Next, we examined whether rapamycin‐induced translational downregulation and the associated reduction in excitatory synaptic proteins could rescue olfactory deficits in VDD mice. Although rapamycin treatment led to a decrease in body weight (Figure S10A, Supporting Information, *P* < 0.01, unpaired t‐test), the brain‐to‐body weight ratio remained unaffected (Figure S10B, Supporting Information, unpaired t‐test). Additionally, general motor function, as measured by the travel distance and speed in the open field, were unaffected; however, rapamycin‐treated mice spent significant less time in the center zone (Figure S10C–E, Supporting Information, *P* < 0.05 for time in the center zone, unpaired t‐test). In the olfactory habituation test, both groups showed a significant decrease in sniffing time between the first and the fourth presentations of isoamyl acetate (Figure 7J, *P* < 0.0001, one‐way repeated ANOVA with Tukey's multiple comparisons post hoc test). However, only the rapamycin‐treated VDD group exhibited a significant increase in sniffing time upon odor switch to 2‐heptanone in the fifth trial (Figure 7J, *P* < 0.0001, one‐way repeated ANOVA with Tukey's multiple comparisons post hoc test). Similar results were observed when the odor sequence was reversed (Figure 7K, *P* < 0.0001, one‐way repeated ANOVA with Tukey's multiple comparisons post hoc test). Moreover, the rapamycin‐treated VDD group demonstrated improved discrimination of structurally similar odor pairs, such as (L)‐limonene and (D)‐limonene, as well as 1‐butanol and 1‐pentanol (Figure 7L,M, *P* < 0.0001, one‐way repeated ANOVA with Tukey's multiple comparisons post hoc test), indicating enhanced olfactory function compared to the saline‐treated group. Importantly, both groups exhibited similar intrinsic preferences for the odor pairs, confirming that rapamycin treatment did not alter odor preference (Figure 7N, unpaired t‐test). Taken together, these findings suggest that mTOR inhibition via rapamycin rescues olfactory deficits in VDD mice by normalizing translation and excitatory synaptic protein expression, further supporting the role of mTOR signaling in mediating the effects of VitD deficiency on synaptic and behavioral function.

### 2.8 Integrated Chromatin Immunoprecipitation Sequencing (ChIP‐seq) Analysis Confirms Direct VDR Binding to Genes Encoding Synaptic Proteins and Translational Machinery Components, Including mTOR Pathway Effectors

**Figure 8:**
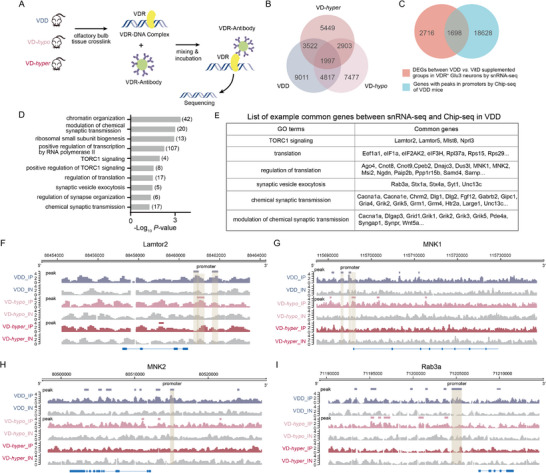
ChIP‐seq reveals that VDR directly binds promoter regions of synaptic and translational target genes. A) Schematic flowchart for VDR ChIP‐seq in OBs from mice fed diets containing three different doses of VitD_3_ (*n* = 2 mice per group). B) Venn diagram of genes with VDR‐bound promoter peaks across VDD, VD‐*hypo*, and VD‐*hyper* groups. C,D) Venn diagram showing the overlap between: DEGs in VDR^+^ Glu3 neurons (snRNA‐seq; VDD vs VitD‐supplemented groups), and genes with VDR promoter peaks (ChIP‐seq; VDD) (C). GO enrichment analysis of overlapping genes (D; *x*‐axis: −log_10_ (*P* value); *y*‐axis: GO terms). E) Representative overlapping genes from key GO terms. F–I) VDR chromatin binding profiles at *Lamtor2* (F), *MNK1* (G), *MNK2* (H), and *Rab3a* (I) (*x*‐axis: genomic coordinates; *y*‐axis: normalized ChIP‐seq signal intensity). Gray shading indicates significant peaks at promoters; tracks show VDR‐specific immunoprecipitation (IP) versus input controls (IN) across three groups (VDD, VD‐*hypo*, and VD‐*hyper*).

We next investigated VitD‐mediated translational and synaptic regulation through VDR‐dependent genomic mechanisms. Given VDR's function as a transcription factor, we performed ChIP‐seq analysis of regulatory sites in OB samples from VDD, VD‐*hypo*, and VD‐*hyper* mice (**Figure** **8**A). Interestingly, there were significant differences in VDR binding peaks at promoter regions across three conditions (Figure 8B; and Table S17, Supporting Information), suggesting VitD‐dependent chromatin modifications. Integration of ChIP‐seq with snRNA‐seq data from VDR^+^ Glu3 neurons identified 1699 genes showing both differential expression between VDD and VitD‐supplemented groups and VDR binding at their promoter regions (Figure 8C). GO analysis demonstrated significant enrichment (*P* < 0.05) among these genes for TORC1 signaling, translational regulation, synaptic vesicle exocytosis, and chemical synaptic transmission (Figure 8D,E; and Table S18, Supporting Information). Specifically, we observed enhanced VDR binding at promoters of mTOR signaling components (e.g., Lamtor2), translational regulators (MNK1/MNK2), and presynaptic machinery molecules (Rab3a) in VDD conditions (Figure 8F–I). These findings were validated by elevated MNK protein levels in synaptic fractions and increased Rab3a transcript levels in VDR^+^ Glu3 neurons from VDD mice (Figures 6 and 7), consistent with potential VDR‐dependent modulation of transcriptional regulation by VitD.

Interestingly, while ChIP‐seq revealed significantly elevated VDR binding peaks in promoter regions in the VD‐*hypo* group (Figure S11A, Supporting Information), this group exhibited the lowest levels of total VDR transcripts, the fewest VDR^+^ Glu3 neurons, and the lowest number of VDR^+^ spots compared to both the VDD and VD‐*hyper* groups (Figure S11B,C, Supporting Information). In contrast, total VDR protein levels remained similar across all three groups (Figure S11D, Supporting Information). These results suggest that varying serum VitD levels exert complex regulatory effects on VDR at both transcriptional and translational levels, which cannot be explained by simplified feedback‐based homeostatic regulation.

## 3 Discussion

VitD emerges as a critical regulator of neuronal activity through mechanisms that converge on synaptic function. While VitD deficiency has long been associated with neurological impairment, our findings reveal its specific role in sensory processing through VDR‐mediated pathways in tufted cells of the OB. We demonstrate that VitD status modulates olfactory function—deficiency impairs odor discrimination while supplementation enhances sensitivity—effects phenocopied by selective VDR knockdown. Importantly, VitD orchestrates dendrodendritic synaptic remodeling through a dual‐mode mechanism: classical genomic regulation via direct VDR binding to synaptic and translational genes, coupled with a newly identified mTOR‐dependent translational control pathway. The rescue of both molecular and behavioral deficits by rapamycin in VitD‐deficient mice highlights the physiological relevance of this VDR‐mTOR axis. These discoveries position VitD as a diet‐responsive regulator that functionally links peripheral nutritional status to the central nervous system synaptic function and sensory processing through integrated transcriptional and translational mechanisms.

**Figure 9:**
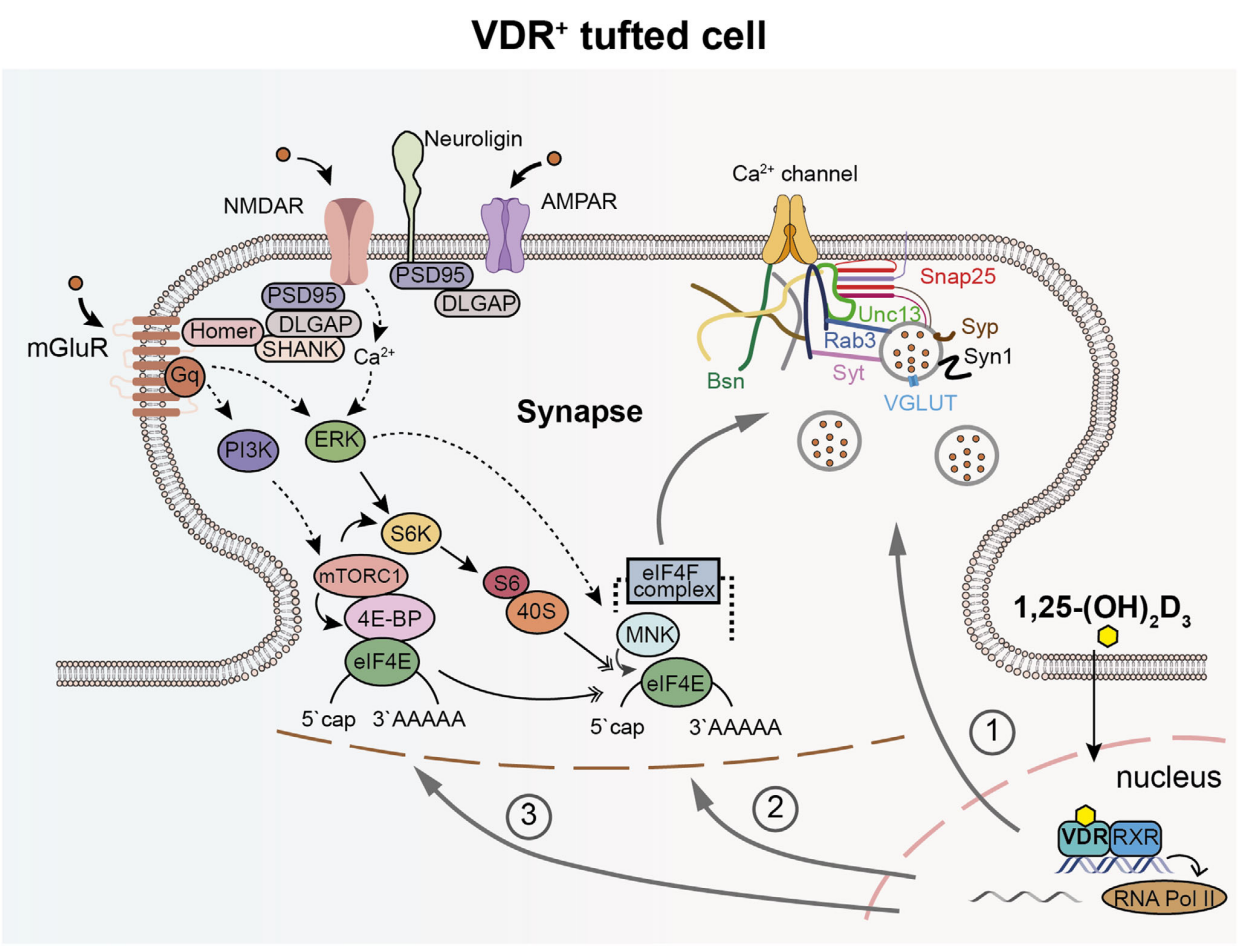
VitD regulates synaptic protein expression through VDR‐mediated transcriptional and translational control mechanisms. The schematic illustrates three molecular pathways by which VitD‐VDR signaling regulates synaptic protein expression: 1) Direct promoter binding to synaptic protein genes (e.g., Rab3a, Syt1; pathway 1); 2) Transcriptional control of translational regulators, including MNKs, initiation/elongation factors, and ribosomal proteins (pathway 2); 3) Modulation of mTOR signaling component (e.g., Lamtor2/5; pathway 3).

VitD regulates synaptic protein expression through interconnected transcriptional and translational mechanisms, revealing a complex regulatory landscape. Our findings demonstrate three primary pathways mediating these effects (**Figure** **9**): First, VitD deficiency directly elevated transcript levels of synaptic proteins (e.g., Rab3a and Syt1) through enhanced promoter binding. Second, we identified VitD‐dependent transcriptional control of translational regulators (e.g., MNKs, initiation/elongation factors, and ribosomal proteins), with deficiency leading to increased translation of synaptic components. This was evidenced by the selective synaptic enrichment of eIF4E, Rps6, and MNK in deficient animals, indicating localized perturbations in protein synthesis machinery.^[^ ^49^ ^]^ Third, VitD modulated transcription of mTOR signaling components (e.g., Lamtor2/5). VitD deficiency increased transcripts of these genes, elevating downstream phosphorylation of S6K and eIF4EBP—a mechanism that likely drives enhanced translation of excitatory synaptic proteins. Complementing these findings, elevated p‐AKT levels in VitD‐deficient conditions suggested additional ERK‐dependent translational activation downstream of enhanced excitatory transmission.^[^ ^50^ ^]^ Together, these mechanisms reveal VitD's multifaceted role in synaptic regulation, integrating transcriptional control with translational modulation through the PI3K/AKT/mTOR axis.

Our study revealed VitD's critical role in maintaining excitatory‐inhibitory balance within OB microcircuits. Deficiency induced disproportionate increases in excitatory synaptic markers (vGlut1, PSD95) relative to inhibitory components, a perturbation that likely disrupts precise gamma/theta oscillations and temporal spike patterns essential for odor discrimination.^[^ ^51^, ^52^, ^53^ ^]^ These synaptic alterations exhibited striking regional specificity, differing substantially from those reported in hippocampal or cortical circuits under both gestational deficiency^[^ ^54^, ^55^ ^]^ and adult supplementation conditions.^[^ ^22^, ^23^ ^]^ Supporting this spatiotemporal specificity, comparative ChIP‐seq analysis demonstrated fundamentally distinct VDR genomic binding patterns in olfactory neurons versus human cell lines,^[^ ^17^, ^56^ ^]^ suggesting cell‐type‐specific mechanisms underlie VitD's varied neural effects.

Several important limitations of our study warrant consideration: First, the structural basis for synaptic protein elevation—whether reflecting increased synapse number or modified release site architecture—requires ultrastructural examination. Second, we cannot yet distinguish cell‐autonomous effects in VDR^+^ tufted cells from secondary network adaptations through synaptic rebalancing. Third, the functional consequences for the synaptic modification in tufted cells remain to be experimentally explored. Addressing these questions will be crucial for understanding how nutritional status shapes neural circuit function through VitD‐dependent mechanisms, with broader implications for sensory processing disorders.

Collectively, our findings establish a functionally specialized subpopulation of tufted cells as a critical VitD‐sensitive node in olfactory circuit regulation, wherein mTOR‐dependent control of synaptic protein translation serves as a key mechanistic link between nutritional state and sensory processing. Beyond advancing fundamental knowledge of nutrient‐sensitive synaptic regulations, these insights highlight novel therapeutic opportunities—whether through dietary optimization, VitD supplementation, or strategic mTOR modulation—as potential stand‐alone or adjunctive interventions in neurodevelopmental and neurodegenerative disorders marked by synaptic dysfunction.

### 3.1 Resource Availability

The structured datasets—including snRNA‐seq, spatial transcriptomics, bulk RNA sequencing—are publicly available through the NCBI repository (BioProjectID: PRJNA1248507). The mass spectrometry proteomics data have been deposited to the ProteomeXchange Consortium (https://proteomecentral.proteomexchange.org) via the iProX partner repository with the dataset identifier PXD062768. Additional raw datasets not deposited in NCBI can be obtained upon request from the corresponding author or accessed via the Mendeley Data repository (https://data.mendeley.com/preview/mfs9283rt9?a=883de594‐374f‐4911‐82c3‐2ce3f4efd2e2).

## 4 Experimental Section

### Mice

Three‐week‐old male C57BL/6J mice were obtained from Hunan SJA Laboratory Animal Co., Ltd. Vglut2‐ires‐Cre (Slc17a6^2(cre)Lowl^/J, Jacksonlab Strain #01 6963) transgenic mice^[^ ^37^ ^]^ were generously provided by Dr. Xu Chun's research group at the Institute of Neuroscience, Chinese Academy of Sciences, Shanghai. Mice were housed in individually ventilated cages under a 12‐h light/12‐h dark cycle, with five mice per cage, and provided ad libitum access to water. Environmental conditions were maintained at a temperature of 22 ± 2 °C and a relative humidity of 55 ± 10%. All experimental procedures were conducted in compliance with the ethical guidelines approved by the Ethics Committee of Hainan Medical University (Approval Numbers: HYLL‐2022‐259 and HYLL‐2024‐173). Unless otherwise specified, mice were fed the standard AIN‐93G diet ad libitum.

### VitD_3_ Supplementation

Mice with gradient VitD_3_ supplementation were established as described before.^[^ ^20^ ^]^ In brief, mice were weaned at P21 and fed customized diets formulated to provide daily VitD_3_ intakes of 0, 500, or 2000 IU kg^−1^ for a minimum of 12 weeks, corresponding to the VDD group, VD‐*hypo* group and VD‐*hyper* group. Six custom diets, based on the standard AIN‐93G formulation with VitD_3_ concentrations of 0, 1.00 × 10^3^, 2.50 × 10^3^, 5.00 × 10^3^, 1.00 × 10⁴, and 2.00 × 10⁴ IU kg^−1^, were obtained from Jiangsu Xietong Bioengineering Co., Ltd. (Nanjing, China).

### Rapamycin Administration

A 20 mg mL^−1^ stock solution of rapamycin was prepared in dimethyl sulfoxide (DMSO, 60313ES60, Yeasen, China) and stored at −80 °C. Prior to injection, the stock solution was diluted to 0.6 mg mL^−1^ using sterile 0.9% saline (BL158A, Biosharp, China). Mice received intraperitoneal injections of rapamycin at 6 mg kg^−1^, three times per week, for six weeks. Control mice received vehicle (DMSO in saline) on the same schedule.

### Measurement of the Serum 25(OH)D

Mice were anesthetized using isoflurane, and ≈500 µL of blood was collected via orbital enucleation. Serum levels of 25(OH)D_2_ and 25(OH)D_3_ were quantified using LC‐MS (API 3200 LC‐MS, AB Sciex) at Daan Gene Company Limited (Guangzhou, China). Total serum 25(OH)D levels were calculated as the sum of 25(OH)D_2_ and 25(OH)D_3_ concentrations.

### Stereotactic Injections

Mice were anesthetized with 2‐3% isoflurane and placed in a stereotactic frame (Reward Co.). Bilateral injections of AAV2‐hSYN‐DIO‐EGFP‐KASH‐WPRES (titer: 2.75 × 10^12^ vg mL^−1^; gift from Dr. Peter Scheiffele, Biozentrum Basel) were performed into the OBs (200–300 nL per site) using the following coordinates relative to bregma: medio‐lateral (ML) ±0.6 mm, anterior‐posterior (AP) +4.5 mm, and dorso‐ventral (DV) −3.0 mm. Mice were processed for immunohistochemistry 2 weeks post‐injection.

For retrograde tracing, either retroAAV‐CMV‐ZsGreen (2.12 × 10^12^ vg mL^−1^; HANBI Co.) or retroAAV‐hSyn‐mCherry‐WPRE‐hGH polyA (5.32 × 10^12^ vg mL^−1^; Brain VTA) was injected into the AON (coordinates: ML ±1.25 mm, AP +2.77 mm, DV ‐3.75 mm). Due to comparable labeling efficiency between viruses (assessed after 3 weeks), datasets were combined for analysis.

VDR knockdown was achieved using AAV2/9‐U6‐sgRNA(VDR)‐EF1α‐EGFP‐WPRE‐pA (2.09 × 10^12^ vg mL^−1^) and AAV2/9‐hSyn‐SaCas9‐3HA‐pA (5.52 × 10^12^ vg mL^−1^; both from Brain VTA). Behavioral testing commenced at 8 weeks post‐injection, while molecular analyses used a separate cohort harvested at 4 weeks.

### Nissl Staining

Mice were anesthetized with isoflurane and transcardially perfused with 4% paraformaldehyde (PFA). After dehydrations, OBs were sectioned at 50 µm thickness using a microtome (Leica CM1950, Wetzlar, Germany). Sections were stained with cresyl violet (Nissl) staining solution (C0117, Beyotime, China), dehydrated through a graded ethanol series, cleared in xylene, and mounted. Images were acquired using a light microscope (Murzider, Z530) and analyzed with Fiji software.

### Immunohistochemistry

Brain sections were blocked for 1 h at room temperature in Tris‐buffered saline (TBS) containing 0.3% Triton X‐100 (30 188 928 Sinopharm Chemical Reagent Co., China) and 10% goat serum (Zhongshan Jinqiao Biotechnology, Beijing, China). Sections were then incubated overnight at 4 °C with primary antibodies diluted in TBS containing 0.3% Triton X‐100 and 5% goat serum. After washing, sections were incubated with secondary antibodies at room temperature for 1 h. Nuclei were counterstained with DAPI (BL105A, Biosharp, China). Images were acquired using a confocal microscope (TCS SP8, Leica, Germany; LSM 900, ZEISS, Germany). The following primary antibodies were used: rabbit anti‐VDR (1:500; Cell Signaling Technology, 12550S); mouse anti‐calbindin D‐28k (1:5000; Swant CB300PUR); mouse anti‐calretinin (1:5000; 6B3, Swant); guinea pig anti‐somatostatin‐28 (1:500; Synaptic Systems, 366 004); mouse anti‐parvalbumin (1:5000; Swant 235); mouse anti‐tyrosine hydroxylase (1:500; Sigma MAB318); guinea pig anti‐S100b (1:200; Synaptic Systems, 287 004). Secondary antibodies used were: goat anti‐rabbit Alexa Fluor 568 (1:500; Invitrogen, A11011); goat anti‐mouse Alexa Fluor 488 (1:500; Invitrogen, A11029); goat anti‐guinea pig Alexa Fluor 488 (1:500; Invitrogen, A11073).

### FISH

Adult mice were anesthetized and decapitated, and brains were rapidly extracted and sectioned at 20 µm thickness using a frozen microtome (Leica CM1950UV, Leica, Germany). RNAscope in situ hybridization was performed according to the manufacturer's protocol (323 100, Advance Cell Diagnostics (ACD), Hayward, CA) and previously described method. Briefly, sections were pretreated and incubated for 30 min on HybEz Hybridization System (321 720, ACD Hayward, CA). Sections were then hybridized with a VDR probe (C1#524 511) in a HybEZ oven (ACD) at 40 °C for 2 h. Negative (320 871, ACD) and positive (320 881, ACD) control probes were used under the same conditions. Nuclei were counterstained with DAPI (BL105A, Biosharp, China). Images were acquired using a confocal microscope (TCS SP8, Leica, Germany) and analyzed using Fiji software. For quantification, two OB sections per mouse (*n* = 3 mice, 6 sections total) were analyzed, with 5–6 microscopic fields captured per section (30–36 images per experiment). DAPI‐positive cells were counted in each anatomical layer (GL, EPL, MCL, and GCL). Cells were considered positive for VDR mRNA expression if they exhibited more than five probe signal spots localized within the nucleus or perinuclear compartment (signal within 2–3 µm^2^).

### RNA Extraction and Real‐Time Quantitative Reverse Transcription PCR (RT‐qPCR)

Total RNA was purified using Eastep Super Total RNA Extraction Kit (Promega LS1040, Shanghai, China). Reverse transcription was performed using the High‐Capacity cDNA Reverse Transcription Kit (Applied Biosystems 4 368 814, Foster City, CA, USA) following the manufacturer's instructions. Quantitative PCR (qPCR) was conducted on a CFX96 real‐time detection system (Bio‐Rad, Hercules, CA, USA) using PowerUp SYBR Green Master Mix (Applied Biosystems A25742, Carlsbad, CA, USA). Primer specificity was confirmed by melting curve analysis and agarose gel electrophoresis. Relative gene expression was calculated using the 2^−ΔΔCT^ method, normalized to GAPDH. All measurements were performed in triplicates.

Primer sequences: VDR: Forward, TCAAACTCTGATCTGTACACCC, Reverse, TGGATGCTGTAACTGACAAGAT; GAPDH: Forward, GGTTGTCTCCTGCGACTTCA; Reverse, TGGTCCAGGGTTTCTTACTCC.

### Protein Extraction and Synaptosome Preparation

Brain tissues were homogenized in RIPA Lysis Buffer (20101ES60, Yeasen, China) supplemented with phenylmethylsulfonyl fluoride (BL507A, Biosharp, China) and a phosphate inhibitor cocktail (BL615A, Biosharp, China). Homogenates were centrifuged at 14,000 rpm for 10 min and the supernatant was collected for protein quantification using the BCA Protein Assay (20201ES76, Yeasen, China).

Synaptosome fraction were isolated using the Syn‐PER Synaptic Protein Extraction Reagent (Thermo Fisher Scientific, Waltham, MA). Tissues were homogenized in 100 µL of Syn‐PER reagent and 1 µL of Halt Protease and phosphatase inhibitor cocktail (Thermo Fisher Scientific). Homogenates were centrifuged at 500×g for 10 min at 4 °C, and the supernatant was further centrifuged at 15000×g for 20 min at 4 °C. The resulting synaptosome pellet was resuspended in 40 µL of Syn‐PER reagent with Halt inhibitor cocktail.

### Wes Simple Western Assays (Protein Simple Wes)

Capillary western blot analysis was performed using the Jess Simple Western system (Bio‐Techne, Minneapolis, MN, USA) as previously described. Capillary cartridges (Protein Simple, SM‐W004‐1), anti‐rabbit detection module chemiluminescence (Protein Simple DM‐001), anti‐mouse detection module chemiluminescence (Protein Simple DM‐002), and EZ standard pack (Protein Simple PS‐ST01EZ‐8) were used according to the manufacturer's instructions. The following antibodies were used (if not mentioned in the immunohistochemistry section): mouse anti‐β‐actin (1:200, Novus, NB600‐501), rabbit anti‐AKT (1:200, Cell Signaling Technology, 4691), rabbit anti‐p‐AKT (1:20, Cell Signaling Technology, 4060), rabbit anti‐eIF4E (1:100, Cell Signaling Technology, 2067), rabbit anti‐eIF4EBP1 (1:100, Cell Signaling Technology, 9644), rabbit anti‐p‐eIF4EBP1 (1:200, Cell Signaling Technology, 2855), rabbit anti‐eIF4EBP2 (1:20, CST, 2845), rabbit anti‐GAPDH (1:5000, Cell Signaling Technology, 5174), mouse anti‐gephyrin (1:4000, Synaptic Systems, 147 111), rabbit anti‐GFP (1:1000; Invitrogen A‐11122), rabbit anti‐mGluR1 (1:500, Cell Signaling Technology, 12 551), rabbit anti‐MNK (1:100, Cell Signaling Technology, 2195), rabbit anti‐p‐MNK (1:20, Cell Signaling Technology, 2111), mouse anti‐mTOR (1:1000, CST, 4517), rabbit anti‐p‐mTOR (1:50, CST, 5536), rabbit anti‐PI3K (1:3000, Cell Signaling Technology, 4249), rabbit anti‐p‐PI3K (1:20, Cell Signaling Technology, 17 366), rabbit anti‐PSD95 (1:100, Cell Signaling Technology, 3409), rabbit anti‐Rps6 (1:100, Cell Signaling Technology, 2217), rabbit anti‐S6K (1:200, CST, 33 475), rabbit anti‐p‐S6K (1:50, CST, 9234), mouse anti‐synapsin1 (1:7000, Synaptic Systems, 106 011), mouse anti‐VGAT (1:20, Synaptic Systems, 131 011), mouse anti‐vGlut1 (1:7000, Synaptic Systems, 135 011), and rabbit anti‐vGlut2 (1:100, Cell Signaling Technology, 16 066).

### RNA Library Construction and Sequencing (Bulk RNA‐seq)

Total RNA of OBs was isolated using the RNeasy Lipid Tissue Mini Kit (Qiagen, Hilden, Germany). RNA quality was assessed by gel electrophoresis and Qubit (Thermo, Waltham, MA, USA). Libraries were prepared using the VAHTS Stranded mRNA‐seq Library Prep Kit for Illumina (Vazyme, China) and sequenced on the Illumina Novaseq 6000 platform. Raw data were processed using Skewer, and quality was assessed with FastQC (v0.11.2). Clean reads were aligned to the reference genome using STAR (2.5.3a). Transcript expression was quantified as FPKM (Fragments Per Kilobase of exon model per Million mapped reads) using StringTie (v1.3.1c). Differentially expressed genes (DEGs) were identified using DEGseq2 (v1.16.1) with a false discovery rate (FDR) < 0.01 and fold change ≥2.

### SnRNA‐seq

OBs from mice were dissected and processed according to the 10X Genomics Chromium protocol. Nuclei were extracted from mouse OB tissue using a protocol adapted from the 10X Genomics Demonstrated Protocol CG00055 (RevA) for sample preparation. Nuclei were loaded onto the 10X Chromium Single Cell Platform at a concentration of 1000 nuclei µL^−1^, following the literature^[^ ^57^, ^58^ ^]^ and the manufacturer's instructions for the Single Cell 3′ library and Gel Bead Kit v.3. Gel beads in emulsion (GEMs) were generated, and barcoding, GEM‐RT clean‐up, cDNA amplification, and library construction were performed following the manufacturer's protocol. Libraries were quantified using Qubit and sequenced on the Illumina NovaSeq 6000 instrument with 150‐base‐pair paired‐end reads.

### Unsupervised Clustering and Visualization

Unsupervised clustering was performed using Seurat package (v2.2) in R. Genes expressed in fewer than two cells were excluded. Cells with >200 genes and <10% mitochondrial genes were retained. Dimensionality reduction was performed using principal component analysis (PCA) based on the top 2000 most variable genes. A k‐nearest neighbor graph was constructed, and clusters were identified using the Louvain Modularity optimization algorithm. Results were visualized using UMAP plots. Cells expressing hemoglobin genes were excluded.

### Marker Gene Identification and Cell‐Type Annotation

Differentially expressed genes for each cluster were identified using Seurat's FindMarkers function with the “bimod” test. Genes with a log_2_ average expression difference >0.585 and *P* < 0.05 were considered marker genes. Clusters were annotated using canonical cell‐type markers.

### CellphoneDB

Cell‐cell communication analysis was performed using CellphoneDB (v5.0.0)^[^^47]^ for Cluster‐3 VDR^+^ Glu3, Cluster‐3 VDR‐ Glu3, and 20 other clusters. Significant interactions (*P* < 0.05) were identified based on normalized cell matrices from Seurat.

### Spatial Transcriptomics Analysis

OB sections (10 µm) from 15‐week‐old mice were processed using the 10X Genomics Visium platform.^[^ ^59^, ^60^ ^]^ These processes included H&E staining, infiltration, and library construction according to the protocol provided by the manufacturer. Libraries were sequenced on the Illumina NovaSeq 6000 platform. The Space Ranger pipeline used to process Visium spatial gene expression data followed the guidelines provided by the manufacturer, including demultiplexing, mm10 mouse reference genome alignment, tissue detection, reference detection, and barcode/UMI counting. High‐quality spots (≥200 genes) were analyzed using Seurat's SCTransform for normalization, PCA for dimensionality reduction, and t‐SNE/UMAP for visualization.

### GO Analyses

GO enrichment analyses were performed using the “clusterProfiler” and “TopGO” R package. *P*‐values were calculated using Fisher's exact test and adjusted using the Benjamini‐Hochberg method, with significance defined as *P* < 0.05.

### GSEA

GSEA was performed using the following workflow: Raw read counts were first normalized to generate a standardized gene expression matrix, which was then imported into the GSEA platform (v4.3.2, https://www.gsea‐msigdb.org/gsea/index.jsp) for enrichment analysis. The method calculates an Enrichment Score (ES) for each predefined pathway based on the ranked distribution of genes across the dataset. Statistical significance of the ES was assessed using a permutation test. The ES values were subsequently normalized across pathways to generate Normalized Enrichment Scores (NES), and significantly enriched pathways were identified after adjusting for multiple testing corrections.

### RCTD

Deconvolution analysis was performed using the RCTD package (v2.0.0).^[^ ^46^ ^]^ SnRNA‐seq data were used to define cell‐type‐specific gene sets, and spatial transcriptomics data were modeled as a Poisson distribution. The expression values for known cell types in the single‐cell data were normalized to their average expression values and summed according to unknown proportional weights, while accounting for the batch random effects from both single‐cell and spatial platforms. A hierarchical statistical model was constructed to estimate the proportions of known cell types and to provide the maximum‐probability estimates for both single and double cell types.

### Quantitative Proteomic Analysis by LC‐MS/MS

Quantitative 4D label‐free whole protein analysis was conducted by Jingjie PTM BioLab (Hangzhou, China). Tissue samples were lysed in buffer containing 8 m urea and 1% protease inhibitor cocktail, followed by sonication on ice (3 cycles). Lysates were centrifuged at 12000 × g for 10 min at 4 °C to remove debris, and the supernatant was collected for protein quantification using a BCA kit. For trypsin digestion, proteins were reduced with 5 mm dithiothreitol at 56 °C for 30 min and alkylated with 11 mm iodoacetamide at room temperature for 15 min in the dark. Peptide precursor ions and their fragments were analyzed using a Bruker timsTOF Pro mass spectrometer (100–1700 m/z) with Parallel Accumulated Serial Fragmentation (PASEF).

Secondary mass spectrometry data were processed using MaxQuant (v1.6.15.0). The *Mus musculus* reference proteome was used for database searching (Mus_musculus_10 090_GCF_0 00001635.26_GRCm38.p6_20 220 323.Fasta; 84985 sequences). A decoy database was included to calculate the false discovery rate (FDR), and a common contamination library was incorporated to account for potential contaminants.

### ChIP‐seq and Data Analysis

ChIP‐seq was performed in Wuhan IGENEBOOK Biotechnology Co.,Ltd according to the previously described protocol .^[^ ^61^ ^]^ In brief, OBs from six 6‐month‐old mice were washed twice in cold PBS buffer and cross‐linked with 1% formaldehyde for 10 min at room temperature and then quenched by addition of glycine (125 mmol L^−1^ final concentration). Afterward, samples were lysed with 50 mm Tris‐HCl (pH 8.0), 10 mm EDTA, 1%SDS, 1×protease inhibitor cocktail, and chromatins were obtained on ice. Chromatins were sonicated to get soluble sheared chromatin (average DNA length of 200–500 bp). Twenty microliters chromatin was saved at –20 °C for input DNA, and 100 ul chromatin was used for immunoprecipitation by anti‐VDR antibodies (Cell Signaling Technology, 12550S). Ten micrograms of antibody was used in the immunoprecipitation reactions at 4 °C overnight. The next day, 30 µL of protein beads was added and the samples were further incubated for 3 h. The beads were next washed once with 20 mm Tris/HCL (pH 8.1), 50 mm NaCl, 2 mm EDTA, 1% Triton X‐100, 0.1% SDS; twice with 10 mm Tris/HCL (pH 8.1), 250 mm LiCl, 1 mMEDTA, 1% NP‐40, 1% deoxycholic acid; and twice with TE buffer 1× (10 mm Tris‐Cl at pH 7.5. 1 mm EDTA). Bound material was then eluted from the beads in 300 µL of elution buffer (100 mm NaHCO3, 1% SDS), treated first with RNase A (final concentration 8 µg mL^−1^) during 6 h at 65 °C and then with proteinase K (final concentration 345 µg mL^−1^) overnight at 45 °C. Immunoprecipitated DNA was used to construct sequencing libraries following the protocol provided by the I NEXTFLEX ChIP‐Seq Library Prep Kit for Illumina Sequencing (NOVA‐5143‐02, Bioo Scientific) and sequenced on Illumina Novaseq 6000 with PE 150 method.

Trimmomatic (version 0.36) was used to filter out low‐quality reads. Clean reads were mapped to the *mouse reference* genome by Bwa (version 0.7.15). Samtools (version 1.3.1) was used to remove potential PCR duplicates. MACS2 software (version 2.1.2) was used to call peaks by default parameters (bandwidth, 300 bp; model fold, 5, 50; *P* value, 0.001). If the summit of a peak located closest to the TSS of one gene, the peak would be assigned to that gene. HOMER (version 4.11) was used to predict motif occurrence within peak. ClusterProfiler (v4.2.2, http://www.bioconductor.org/packages/release/bioc/html/clusterProfiler.html) in R package was employed to perform GO and KEGG enrichment analysis. The GO and KEGG enrichment analysis were calculated using hypergeometric distribution with a *P* value cutoff of 0.05.

### Open Field Test

Mice were placed in a 50 × 50 × 50 cm chamber for 10 min, and their activity was recorded using the VisuTrack animal acquisition and analysis platform (Shanghai Xinruan). The central area was defined as the central quarter of the chamber floor, with the remaining area designated as the edge. Movement traces were analyzed using SuperMaze software (v3.0, Shanghai Xinruan Information Technology Co., Ltd.). The experimental setup was enclosed with curtains, and infrared lighting‐maintained illumination at 15 lux.

### Olfactory Habituation‐Dishabituation Test and Fine Discrimination Test

Olfactory tests were conducted in a 30 × 20 × 15 cm cage containing ≈0.5 cm of irradiated woodchip bedding. For the habituation‐dishabituation test, mice were acclimated to the cage for 30 min. A sterile cotton swab soaked in 0.2% isoamyl acetate (Solarbio, M8040; Xilong Scientific, 123‐92‐2) diluted in mineral oil was presented for 2 min, followed by a 2 min rest period. This procedure was repeated four times (habituation phase). In the fifth trial, a swab soaked in 0.2% 2‐heptanone (Sigma‐Aldrich, H810890) was presented. For the reversal test, 0.2% 2‐heptanone was presented in four trials, followed by 0.2% isoamyl acetate in the fifth trial. Sniffing time, defined as the duration the mouse's nose was within 2 cm of the swab, was recorded manually.

For the fine discrimination test, 0.2% (L)‐limonene (Sigma‐Aldrich, 218 367) or 1‐butanol (Westlink Science, 71‐36‐3) was presented in the first four trials, followed by 0.2% (D)‐limonene (Sigma‐Aldrich, 183 164) or 1‐pentanol (Westlink Science, 71‐41‐0) in the fifth trial.

### Olfactory Preference Test

Mice were acclimated to the test cage for 30 min. Odor pairs [(L)‐limonene and (D)‐limonene; 1‐butanol and 1‐pentanol] were presented simultaneously via cotton swabs for 5 min. The time spent sniffing each odor was recorded. The order of odor presentation was randomized for each test.

### Statistical Analysis of Data

Statistical analyses were performed using GraphPad Prism (v10.0.2). Normality was assessed using the Kolmogorov‐Smirnov or Shapiro‐Wilk tests. Data were presented as mean ± SEM if otherwise stated. Comparisons between two groups were analyzed using an independent sample t‐test (normal distribution) or Mann‐Whitney U test (non‐normal distribution). For multiple groups with normal distribution, one‐way ANOVA followed by Tukey's post hoc test was used. Within‐subject comparisons across three or more conditions utilized one‐way repeated measures ANOVA with Tukey's post‐hoc test. Each data point represents an individual sample or animal. Statistical significance thresholds were set at *P < 0.05, **P < 0.01, ***P < 0.001, and ****P < 0.0001.

### Ethics Approval

All animal experiments were performed according to the Ethics Committee of Hainan Medical University (HYLL‐2022‐259 and HYLL‐2024‐173) and conformed to the ethical principles of welfare of laboratory animals.

## Conflict of Interest

The authors declare no conflict of interest.

## Author Contributions

R.P.C., C.R.H., Y.X.S., and P.W.B. contributed equally to this work and should be considered as the co‐first author. X.L. and X.W. conceived and designed the study. X.L., R.P.C., C.R.H., and Y.X.S. wrote the manuscript. R.P.C. and C.R.H. jointly performed multi‐omics data analysis and conducted quantitative *wes* validation experiments. C.R.H. performed the data analysis of ChIP‐seq. R.P.C. carried out olfactory behavior tests and VDR knockdown experiments, with assistance from B.Q.S., and performed rapamycin treatment studies. Y.X.S. executed FISH and immunohistochemistry assays, along with snRNA‐seq and spatial transcriptomics experiments. P.W.B. established the dietary VitD mouse model and performed bulk RNA sequencing and proteomics analyses. H.M.H. performed the stereotactic injection, and contributed to the data analysis and figure preparation. Y.D. and Z.Q.L. participated in establishing the VitD dietary mouse model and provided technical assistance. All authors critically reviewed and approved the final manuscript.

## Declaration of Generative AI and AI‐Assisted Technologies in the Writing Process

During the preparation of this work the authors used deepseek‐ai/DeepSeek‐V3 in order to improve the language and readability. After using this tool/service, the authors reviewed and edited the content as needed and take full responsibility for the content of the publication.

## Supporting information



Supporting Information

Supplemental Tables 1–18

## Data Availability

The data that support the findings of this study are openly available in iProX at https://proteomecentral.proteomexchange.org, reference number 62768.
