# Supplementary material for: Vitamin D Regulates Olfactory Function via Dual Transcriptional and mTOR‐Dependent Translational Control of Synaptic Proteins
Source: Adv Sci (Weinh). 2025 Dec 16;13(13):e07181. doi: 10.1002/advs.202507181 (PMC12955992; doi:10.1002/advs.202507181)
Supplement: Supplementary file 1 — Supporting Information [file ADVS-13-e07181-s001.docx]

**
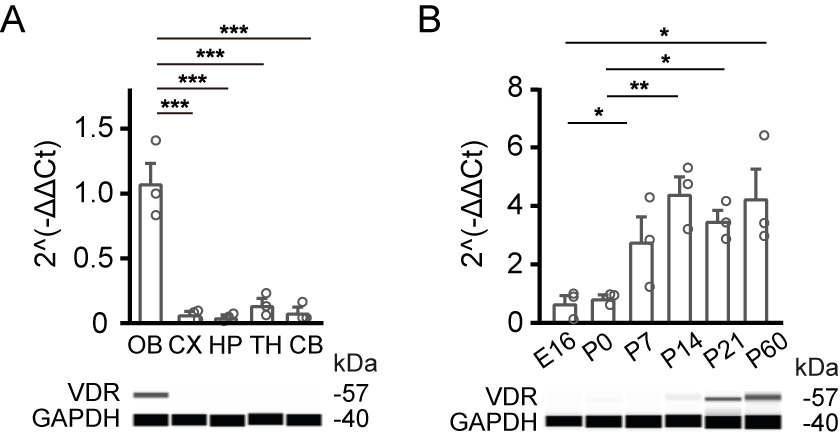
**

**Figure S1. Spatiotemporal expression patterns of VDR mRNA and protein in mouse brain regions and across development.**

**A.** Upper panel*:* relative VDR mRNA expression across adult mouse brain regions (OB: olfactory bulb; CX: cortex; HP: hippocampus; TH: thalamus; CB: cerebellum; *n* = 3 mice). Lower panel: *wes* image showing VDR protein expression (*n* = 1 mouse). **B.** Upper panel*:* developmental trajectory of VDR mRNA expression in OB (E16 to P60; *n* = 3 mice). Lower panel: *wes* image showing VDR protein expression (*n* = 1 mouse/age group). Symbols = biological replicates; bars = mean ± SEM; one-way ANOVA followed by Tukey’s multiple comparisons. Significance levels: **P* < 0.05, ***P* < 0.01, ****P* < 0.001, *****P* < 0.0001.


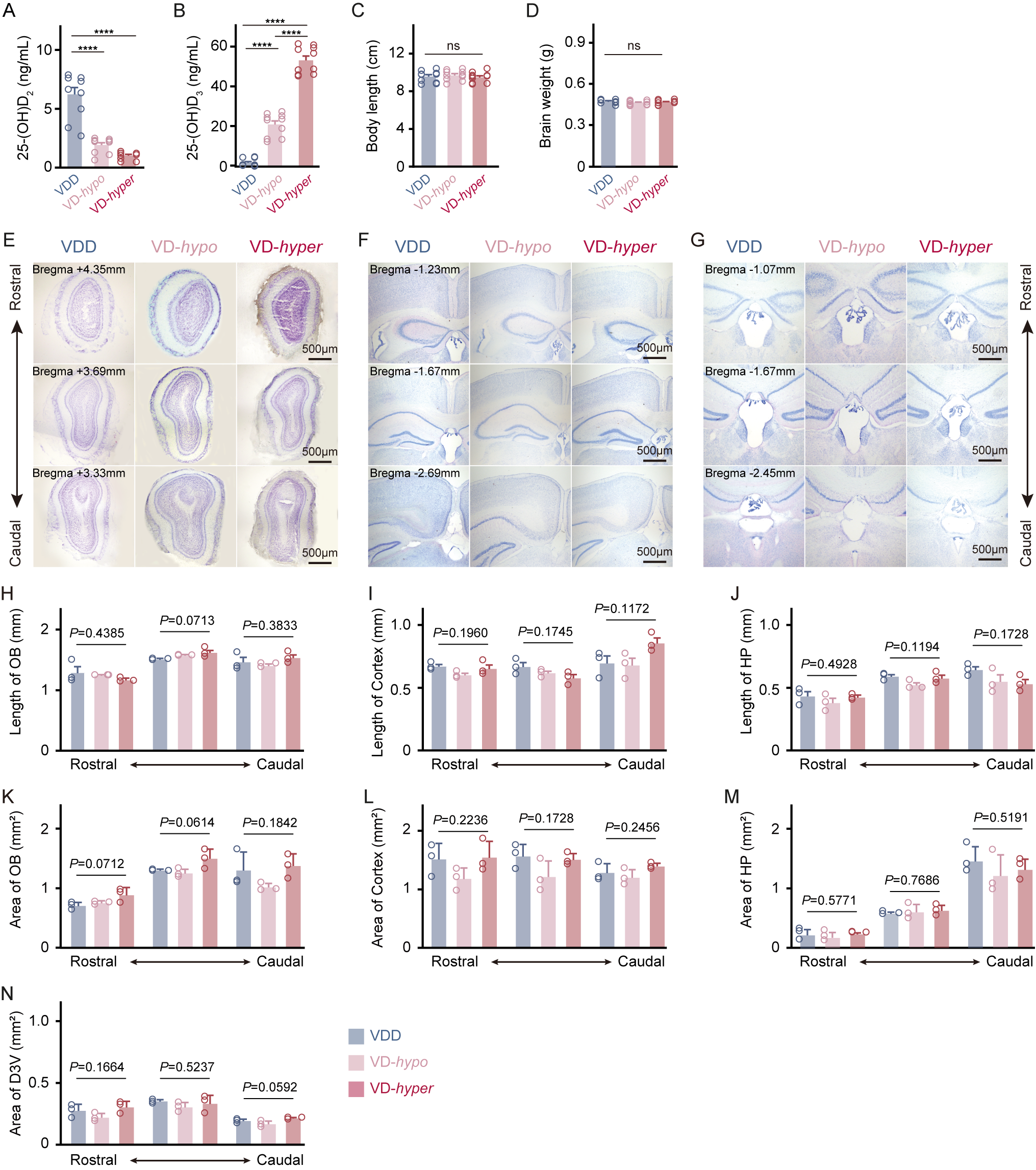


**Figure S2. Varied dietary VitD levels since childhood do not change brain morphology in mice.**

**A-D.** Serum levels of 25-(OH)D_2_ (**A**), 25-(OH)D_3_ (**B**), body length (**C**), and brain weight (**D**) in mice supplemented with varying doses of VitD_3_ since childhood (*n* = 10 mice/group). **E-G.** Representative Nissl-stained coronal sections showing: OB (E), cortex and hippocampus (F), and third ventricle (G) from mice supplemented with different doses of VitD_3_. For each region, three coronal sections from rostral to caudal sides are presented, with bregma coordinates indicated. Scale bar: 500 µm. **H-N.** Quantification of length (**H**), and area (**K**) of OB; length (**I**), and area (**L**) of cortex; length (**J**), and area (**M**) of hippocampus; and area of the third ventricle (**N**) in mice supplemented with varying doses of VitD_3_ (*n* = 3 mice/group). Symbols = biological replicates; bars = mean ± SEM; one-way ANOVA followed by Tukey’s multiple comparisons. Significance levels: **P* < 0.05, ***P* < 0.01, ****P* < 0.001, *****P* < 0.0001; ns: not significant.

**
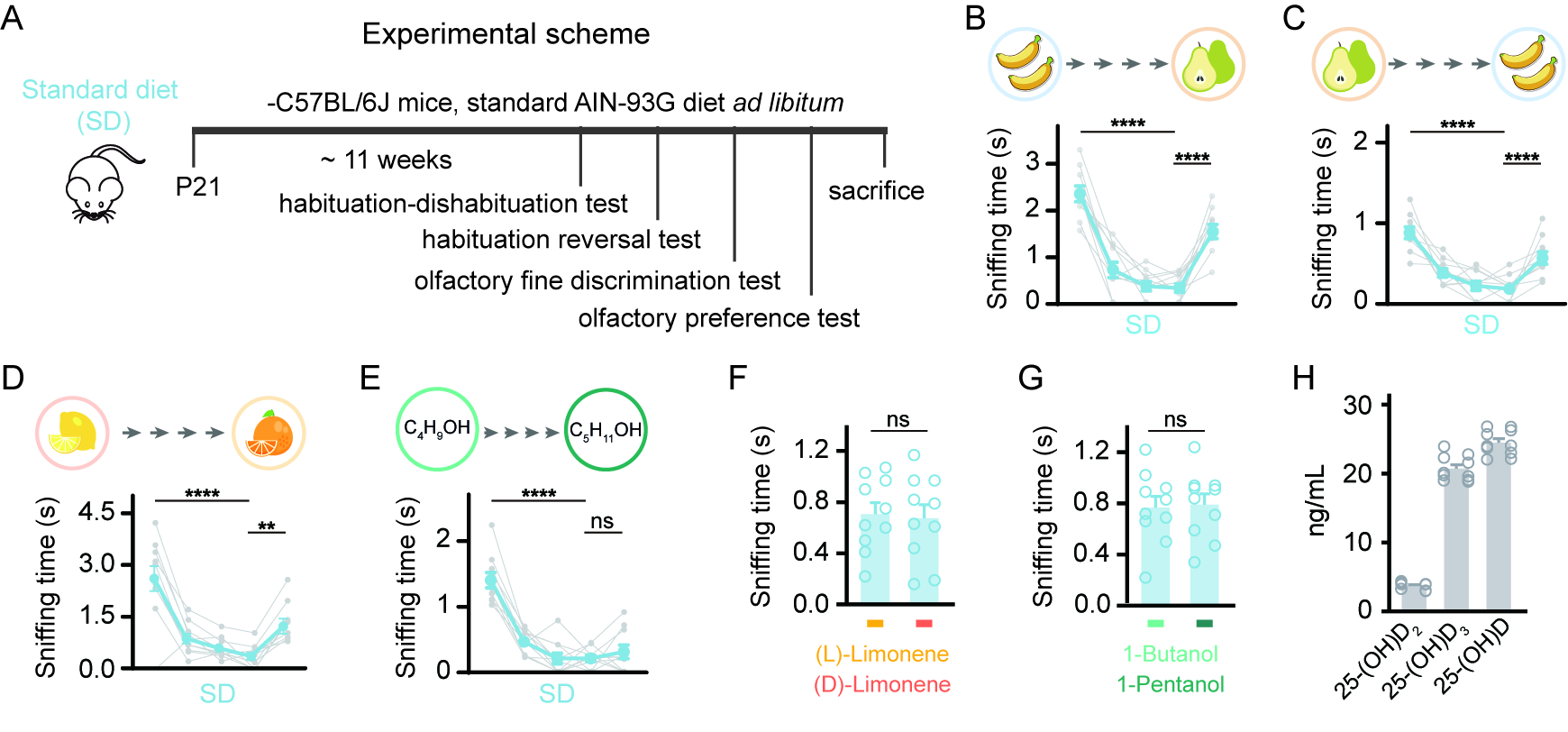
**

**Figure S3. Olfactory function assessment in mice maintained on standard AIN-93G diet (SD group).**

**A.** Experimental timeline: Mice were fed AIN-93G diet ad libitum from P21 for 11 weeks prior to behavioral testing. **B-E.** Sniffing time in olfactory habituation-dishabituation (**B, D, E**) and habituation reversal (**C**) test (one-way ANOVA with Tukey’s multiple comparisons). Tested odor pairs: isoamyl acetate vs. 2-heptanone (**B, C**), (L)-limonene vs. (D)-limonene (**D**), and 1-butanol vs. 1-pentanol (**E**). **F-G.** Olfactory preference test for (L)-limonene vs. (D)-limonene (**F**) and 1-butanol vs. 1-pentanol (**G**) (unpaired t-test). **H.** Serum levels of 25-(OH)D_2_, 25-(OH)D_3_, and 25-(OH)D in 18-week-old SD mice. Symbols = biological replicates; bars = mean ± SEM; *n* = 10 mice. Significance levels: **P* < 0.05, ***P* < 0.01, ****P* < 0.001, *****P* < 0.0001; ns: not significant.

**
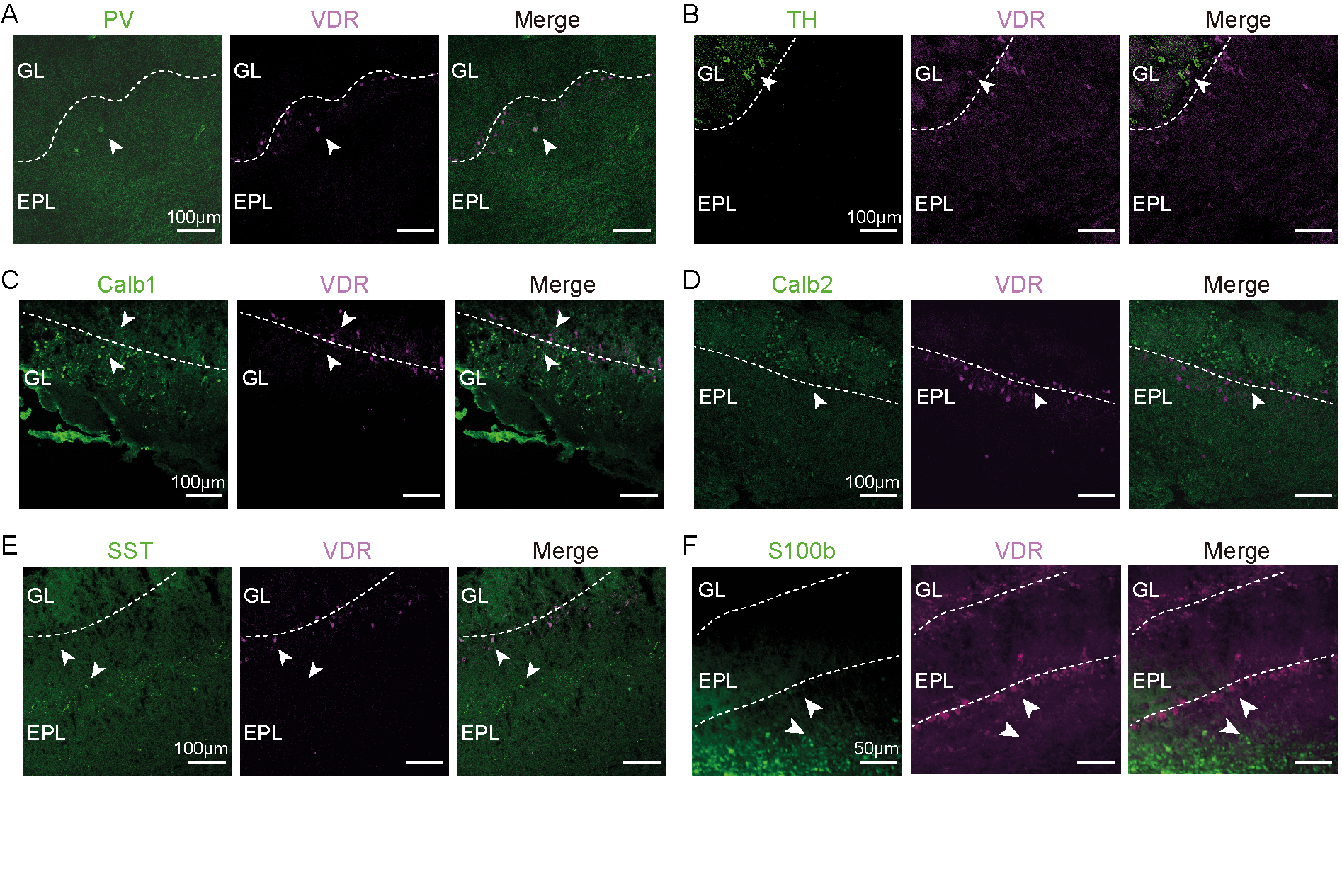
**

**Figure S4. Cell-type-specific expression of VDR in the mouse OB.**

**A-B**. Immunohistochemistry showing colocalization of parvalbumin (PV, green) (**A**) or tyrosine hydroxylase (TH, green) (**B**) with VDR (purple). White arrowheads indicate neurons co-expressing marker proteins. **C-F**. Representative immunohistochemistry images showing that VDR does not co-localize with calbindin (Calb1, **C**), calretinin (Calb2, **D**), somatostatin (SST, **E**), and S100b (**F**) in the OB (indicated by arrowheads). Purple indicates VDR staining. Scale bar: 100 µm (A-E); scale bar: 50 µm (F).


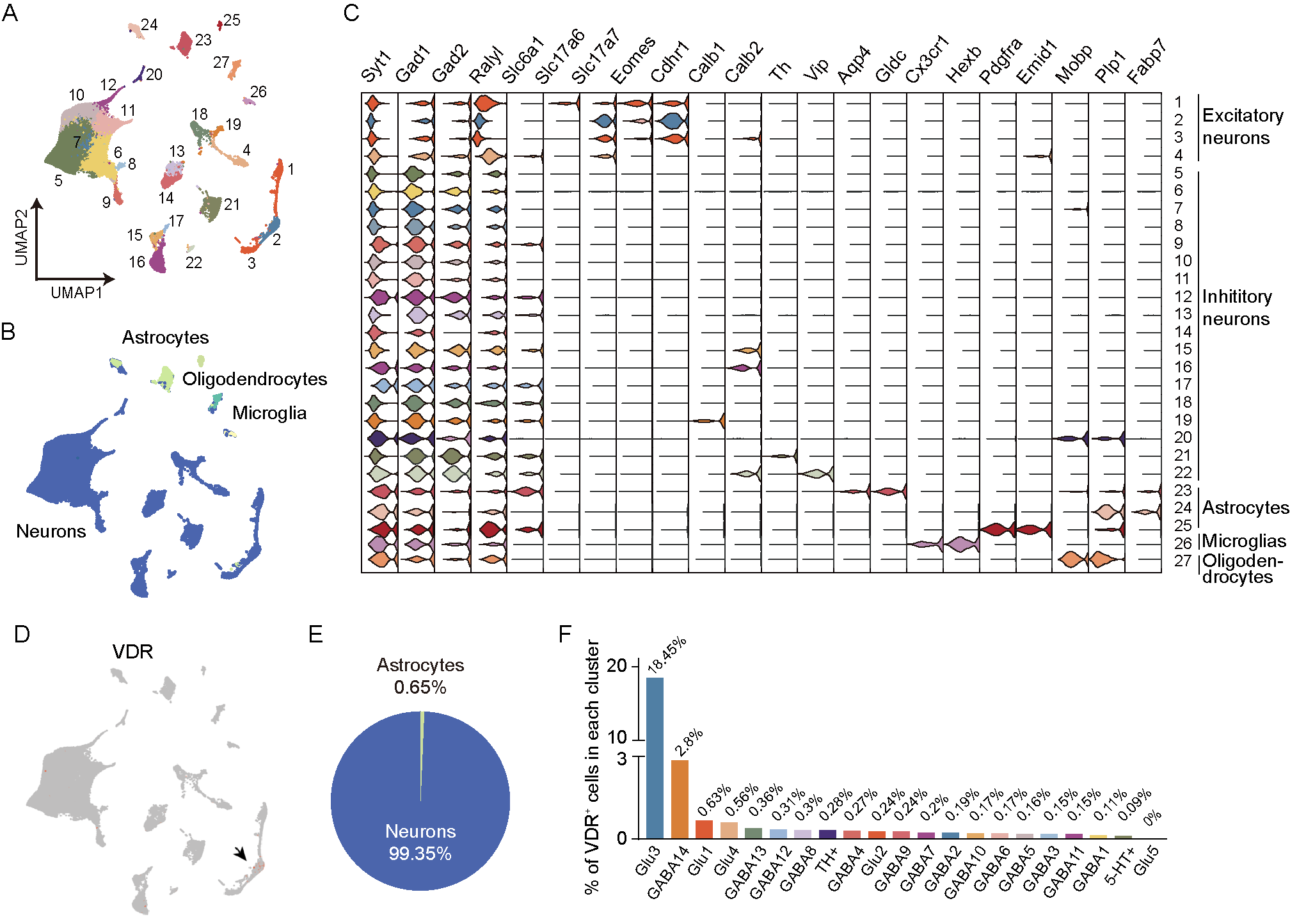


**Figure S5. SnRNA-seq reveals cell-type-specific VDR expression patterns in the OB.**

**A.** UMAP plot showing all identified cell clusters. **B.** UMAP plot colored by annotated cell types. **C.** Violin plot displaying normalized expression levels of canonical marker genes (columns) across 27 cellular clusters (rows). **D.** UMAP plot depicting VDR+ neurons (red). Arrowhead indicates an excitatory neuronal cluster with high VDR expression. **E.** Cellular composition analysis showing neuronal populations account for 99.35% of VDR-expressing cells. **F.** VDR expression frequency among neuronal clusters, revealing highest prevalence in Glu3 neurons (18.45%). Data pooled from n = 6 mice (Two mice per dietary VitD_3_ supplementation group).


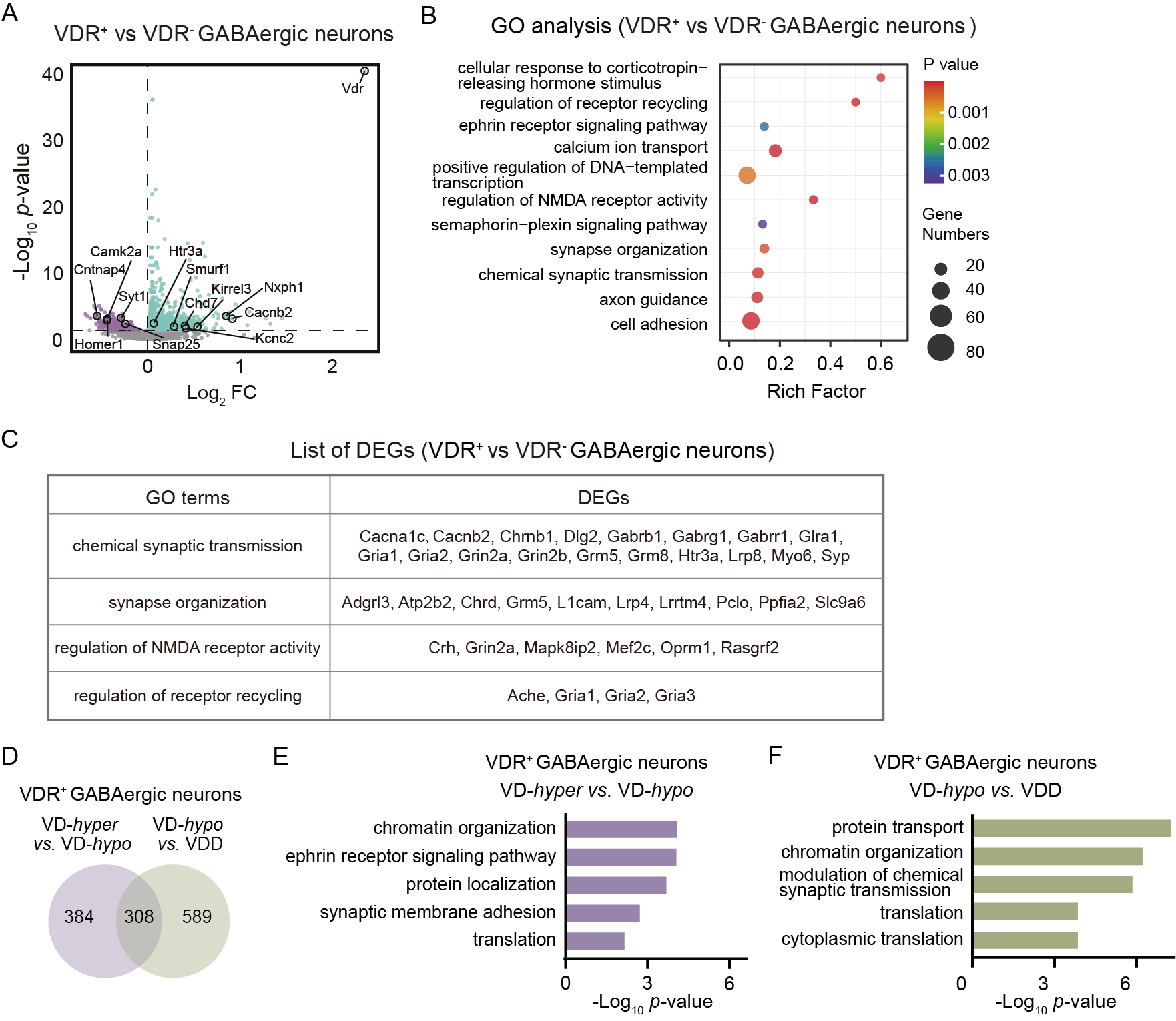


**Figure S6. DEGs and GO enrichment analysis for GABAergic neurons in snRNA-seq data.**

**A.** Volcano plot illustrating DEGs between VDR^+^ and VDR^-^ GABAergic neurons. *x*-axis: log_2_(fold change); *y*-axis: −log_10_ (*P* value). **B.** GO annotation of DEGs between VDR^+^ and VDR^-^ GABAergic neurons. *x*-axis: rich factor; *y*-axis: GO terms; circle size: DEG count; color: *P* value. **C.** List of example DEGs in selected GO terms between VDR^+^ and VDR^-^ GABAergic neurons. **D.** Venn diagram quantifying overlap in DEGs from VDR⁺ GABAergic neurons in comparisons between VD-*hyper* vs. VD-*hypo* and VD-*hypo* vs. VDD. **E-F.** GO annotation of DEGs in VDR⁺ GABAergic neurons comparing VD-*hyper* vs. VD-*hypo* (**E**) and VD-*hypo* vs. VDD (**F**). *x*-axis: −log_10_ (*P* value); *y*-axis: GO terms.


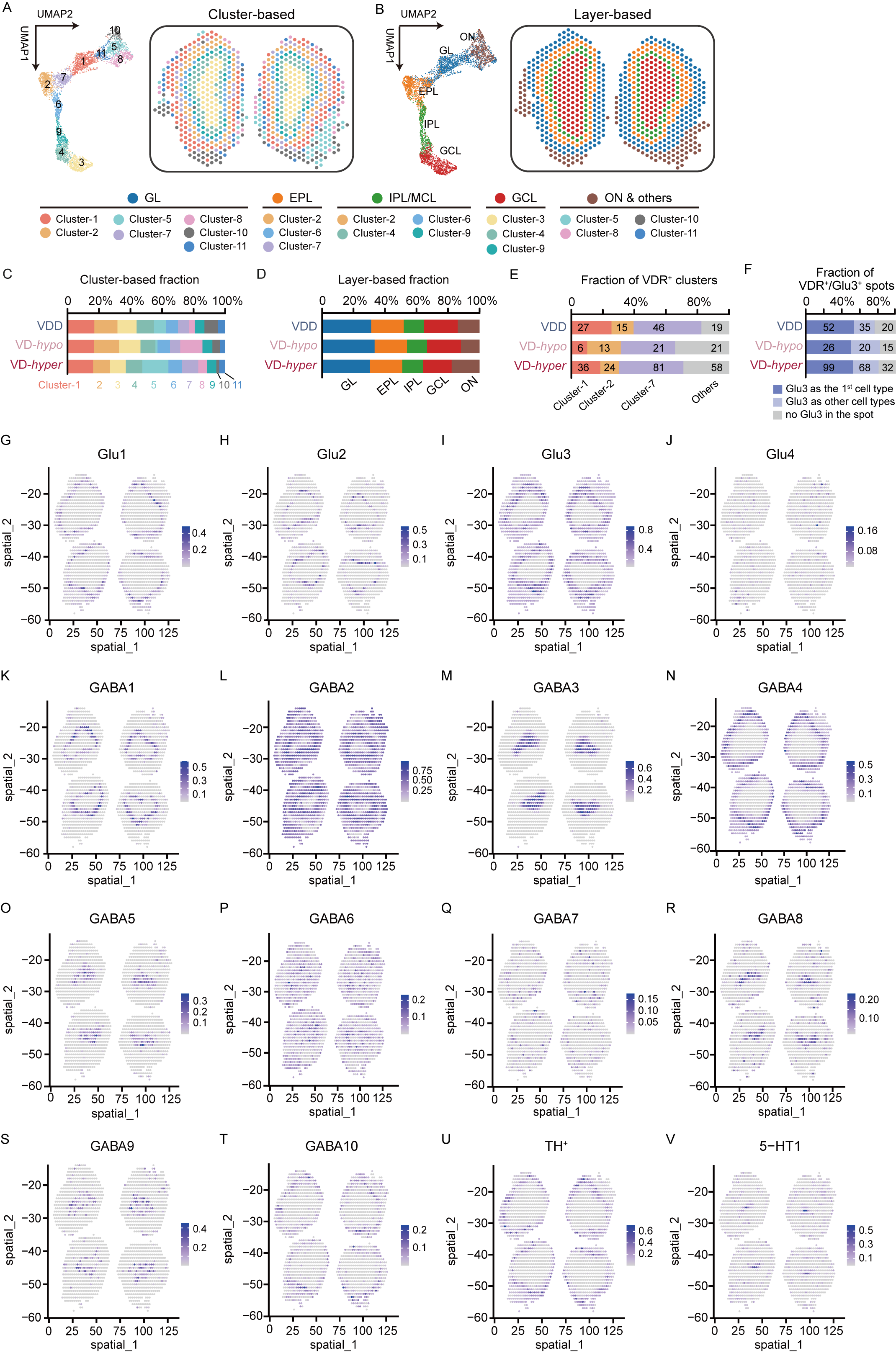


**Figure S7. Spatial transcriptomic profiling of OB sections from mice fed a gradient of VitD_3_.**

**A.** UMAP plot (left) of 11 clusters classified based on spatial transcriptomic data (right; *n* = 1 mouse/group). **B.** UMAP plot (left) of the 6 layer-based clusters classified from spatial transcriptomic data (right). **C-D.** Quantitative distribution analysis of transcriptomic spots according to cluster-based (**C**) and layer-based (**D**) classification. **E.** Number and fraction of VDR^+^ spots according to cluster-based classification in three groups of mice (VDD, VD-*hypo*, and VD-*hyper*). **F.** Cell-type attribution of VDR^+^ spots via RCTD analysis: Glu3-dominant as the primary cell type, Glu3-containing as other cell type, or Glu3-absent. Stacked bars depict the three category proportions with each case presented in the corresponding bar. **G-V.** Spatial distribution maps demonstrating RCTD-classified cell type localization: Glutamatergic subsets: Glu1-4 (**G-J**); GABAergic subsets: GABA1-10 (**K-T**); other neuronal types: TH^+^ (**U**), 5-HT1 (**V**).

**
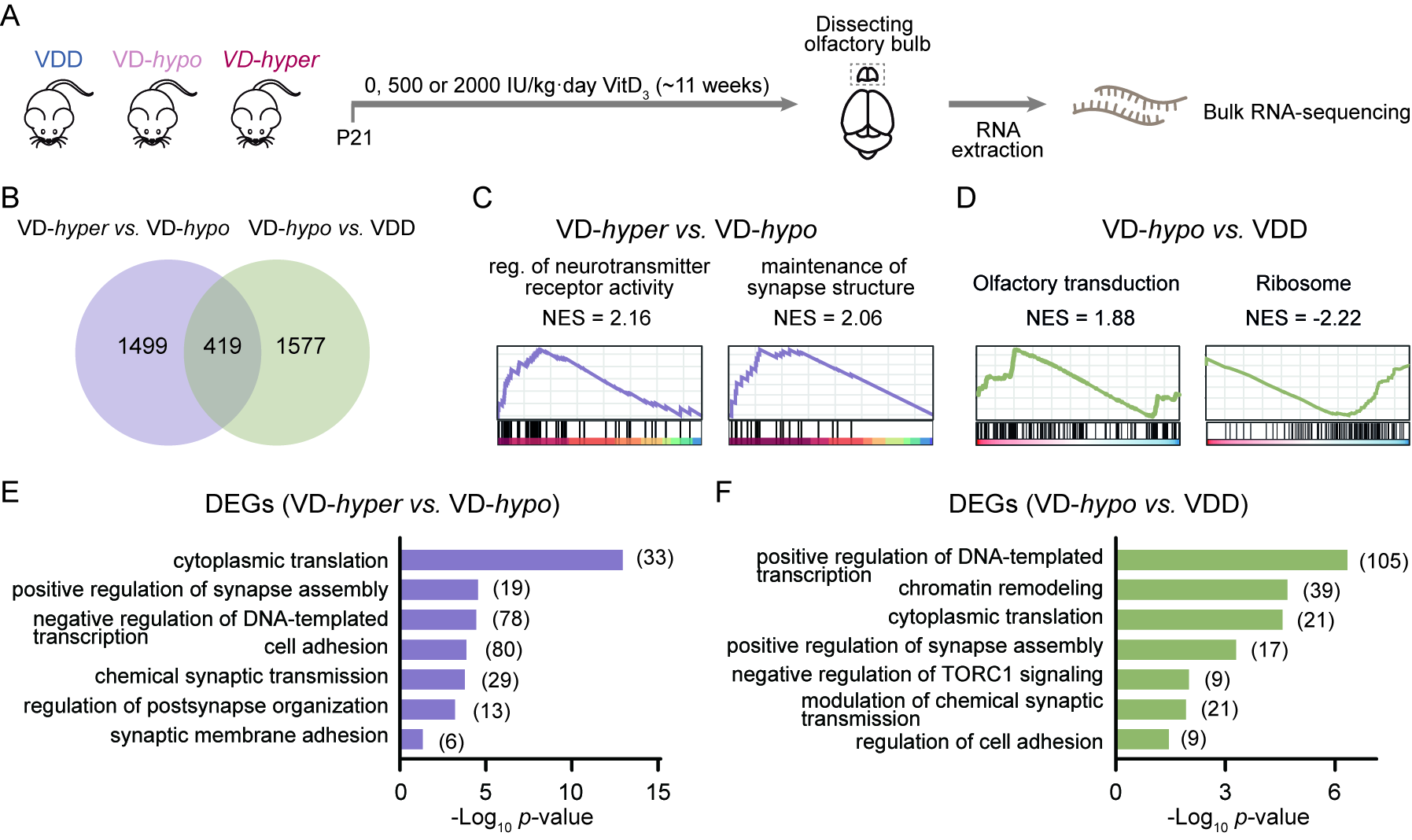
**

**Figure S8. Bulk RNA-sequencing reveals VitD_3_ dose-dependent transcriptional regulation in the OB.**

**A.** Experimental design for bulk RNA-seq profiling of OBs from mice fed three VitD_3_ doses (*n* = 3 per group). **B.** Venn diagram identifying overlapping DEGs between VD-*hyper* vs. VD-*hypo* and VD-*hypo* vs. VDD comparison groups. **C-D.** GSEA highlights dose-responsive pathways: changes in neurotransmitter receptor activity (NES = 2.16) and synaptic structure maintenance (NES = 2.06) in VD-*hyper* vs VD-*hypo* (**C**); changes in olfactory conduction activation (NES = 1.88) and ribosome suppression (NES = -2.22) in VD-*hypo* vs VDD (**D**). **E-F.** GO analysis of DEGs comparing VD-*hyper* vs. VD-*hypo* (**E**) and VD-*hypo* vs. VDD (**F**). *x*-axis: −log_10_ (*P* value); *y*-axis: GO terms. Statistical analysis conducted using standard pipelines for RNA-seq data, with normalization and multiple testing correction applied. NES values indicate normalized enrichment scores from GSEA.


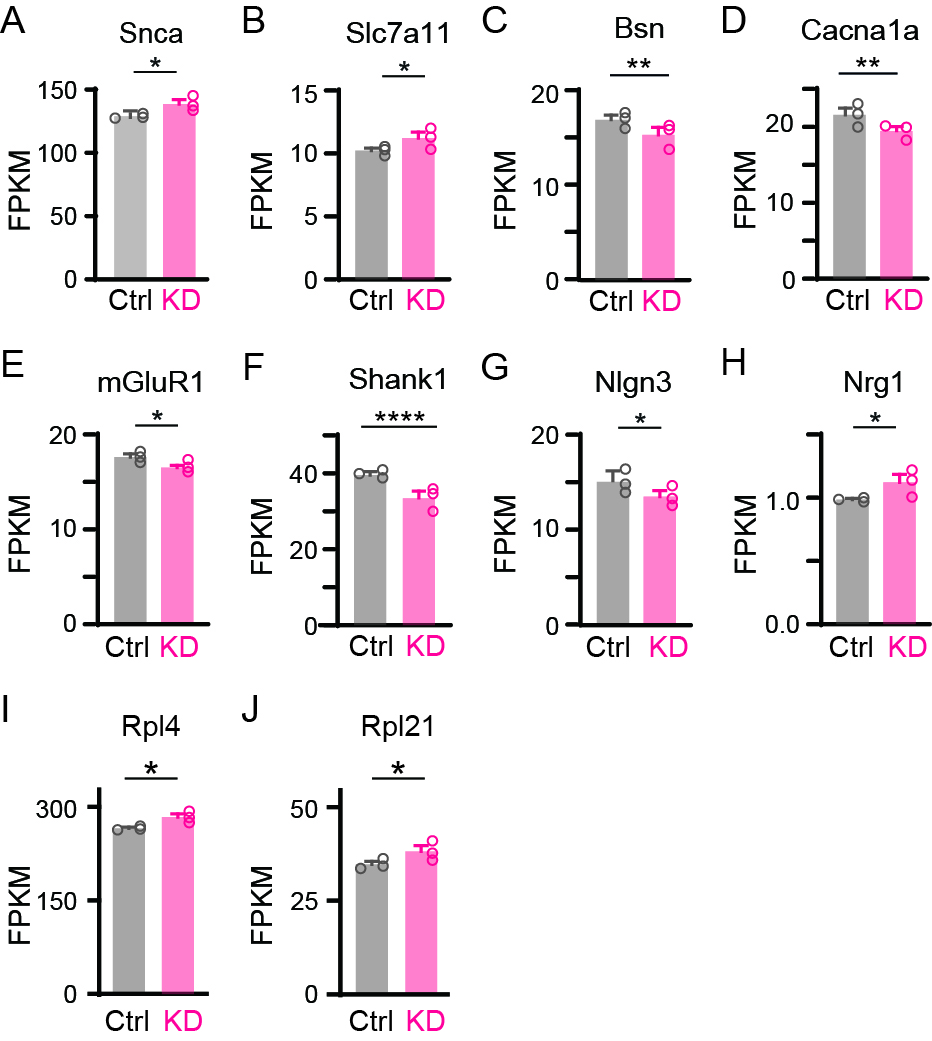


**Figure S9. VDR knockdown perturbs synaptic and translational genes in the OB.**

**A-J.** Relative mRNA expression of *Snca* (**A**), *Slc7a11* (**B**), *Bsn* (**C**), *Cacna1a* (**D**), *mGluR1* (**E**), *Shank1* (**F**), *Nlgn3* (**G**), *Nrg1* (**H**), *Rpl4* (**I**), and *Rpl21* (**J**) in control (Ctrl) and VDR-KD mice (*n* = 3 mice/group). Statistical analysis conducted using standard pipelines for RNA-seq data, with normalization and multiple testing correction applied). Symbols = biological replicates; bars = mean ± SEM. Significance levels: **P* < 0.05, ***P* < 0.01, ****P* < 0.001, *****P* < 0.0001; ns: not significant. FPKM: Fragments per kilobase million.


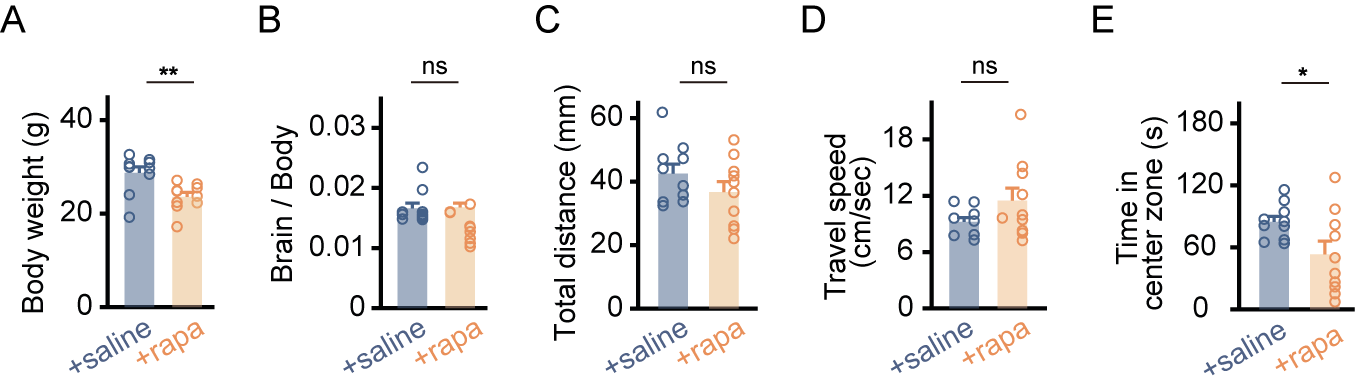


**Figure S10. Physiological and behavioral effects of rapamycin treatment in mice.**

**A-B.** Body weight (**A**) and brain-to-body weight ratio (**B**) comparing rapamycin-treated (rapa) and saline-treated control mice. C-E. Total distance traveled (**C**), average travel speed (D), time to enter the central area (E) of two groups of mice (saline and rapa) in the open field test. Symbols = biological replicates; bars = mean ± SEM; *n* = 10 mice/group; unpaired t-test. Significance levels: **P* < 0.05, ***P* < 0.01, ****P* < 0.001, *****P* < 0.0001; ns: not significant.


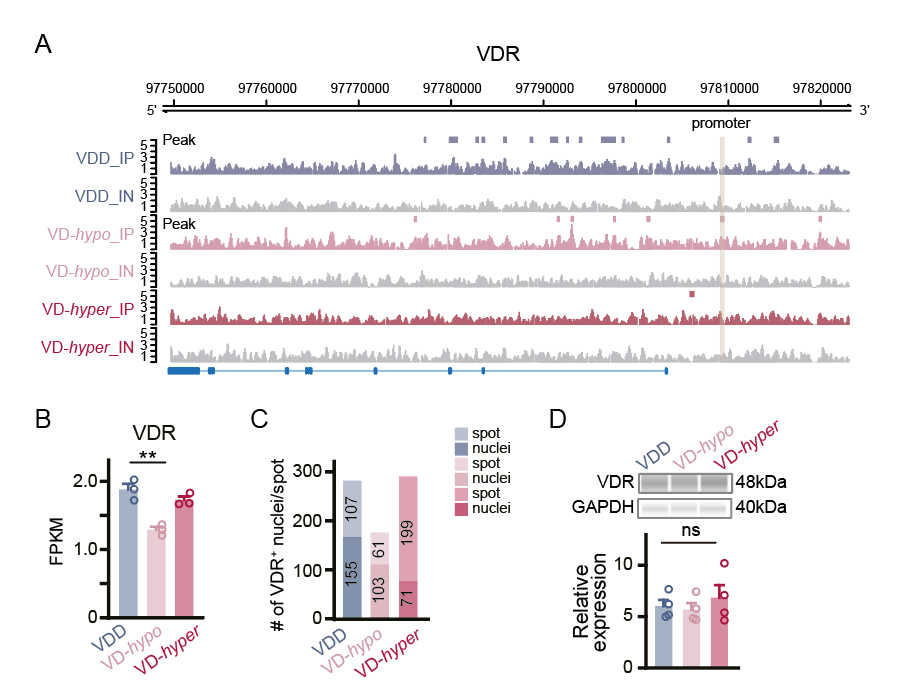


**Figure S11. VDR binding patterns and expression profiles in the OB from mice fed a gradient of VitD3.**

**A.** ChIP-seq reveals VDR binding peaks at promoter region of VDR itself in OBs across VDD, VD-*hypo*, and VD-*hyper* mouse groups. x-axis: genomic coordinates; y-axis: normalized signal of VDR immunoprecipitation (IP) vs. input (IN) across three groups. Gray shading indicates significant peaks at promoters (*n* = 2 mice/group). **B.** VDR mRNA expression quantified by bulk RNA-seq in VDD, VD-*hypo*, and VD-*hyper* groups (*n* = 3 mice/group). **C.** Number of VDR⁺ cells detected by snRNA-seq (light color) and spatial transcriptomics (dark color). **D.** Protein-level validation of VDR expression: representative *wes* image (top) and quantification (bottom) in the OB of VDD, VD-*hypo*, and VD-*hyper* groups (*n* = 4 mice/group). Symbols = biological replicates; bars = mean ± SEM. Significance levels: **P* < 0.05, ***P* < 0.01, ****P* < 0.001, *****P* < 0.0001; ns: not significant.
